# Population-scale identification of differential adverse events before and during a pandemic

**DOI:** 10.1038/s43588-021-00138-4

**Published:** 2021-10-05

**Authors:** Xiang Zhang, Marissa Sumathipala, Marinka Zitnik

**Affiliations:** 1 Department of Biomedical Informatics, Harvard Medical School, Harvard University, Boston, MA USA; 2 Broad Institute of MIT and Harvard, Cambridge, MA USA; 3 Harvard Data Science Initiative, Cambridge, MA USA

**Keywords:** Adverse effects, Drug development, Epidemiology, SARS-CoV-2, Public health

## Abstract

Adverse patient safety events, unintended injuries resulting from medical therapy, were associated with 110,000 deaths in the United States in 2019. A nationwide pandemic (such as COVID-19) further challenges the ability of healthcare systems to ensure safe medication use and the pandemic’s effects on safety events remain poorly understood. Here, we investigate drug safety events across demographic groups before and during a pandemic using a dataset of 1,425,371 reports involving 2,821 drugs and 7,761 adverse events. Among 64 adverse events identified by our analyses, we find 54 increased in frequency during the pandemic, despite a 4.4% decrease in the total number of reports. Out of 53 adverse events with a pre-pandemic gender gap, 33 have seen their gap increase with the pandemic onset. We find that the number of adverse events with an increased reporting ratio is higher in adults (by 16.8%) than in older patients. Our findings have implications for safe medication use and preventable healthcare inequality in public health emergencies.

## Abstract (web summary)

An algorithmic approach is developed to analyze large-scale patient safety data and remove the confounders of reporting trajectory and drug inference. Such an approach can be effectively used to investigate demographic disparities of drug safety and to identify at-risk patients during a pandemic.

## Main

Adverse events from medications^1–4^ are undesirable experiences associated with pharmacotherapy that accounted for over 110,000 deaths in the United States alone in 2019. Despite urgent implications^5–7^, it remains largely unknown how a nationwide pandemic (such as COVID-19)^8–14^ can influence patient safety and what inequalities in diverse patient populations can be exacerbated more than expected had the pandemic not occurred. Furthermore, intricate dependencies between the pandemic’s characteristics, drugs’ mechanisms of action and patient demographics^15,16^ present a unique challenge for understanding patient safety during a public health emergency. Addressing this challenge can inform drug prescription, improve patient safety by identifying individuals at high risk for adverse events, and enable comparison between various health emergencies to unveil the disruptive nature of a public health crisis and inform health policy. To this end, algorithmic approaches are needed to unveil how patient safety has changed with the onset of the pandemic and to compare patient safety to its pre-pandemic levels across patient groups and the entire range of approved drugs and adverse drug events. Previous studies on adverse events have focused on laboratory environments, molecular characterization of drugs and target proteins and limited clinical trial observations^17–19^. Studies of patient safety during a pandemic are restricted to a very limited set of drugs and adverse reactions and are stifled by the small number of adverse event reports and narrow time ranges^20^. Also, such narrowly focused analyses can be confounded by historical biases in adverse event reporting and by mixing population groups that differ in their relative risks for clinical events.

In this Article, we develop an algorithmic approach to systematically investigate adverse events associated with medication use and how they change during a pandemic. Although we conduct analyses in the context of COVID-19—a threatening global pandemic^21,22^—our approach can generalize to other nationwide public health emergencies. Using a patient safety dataset of 1,425,371 adverse event reports spanning seven years (January 2013 to September 2020) and involving 2,821 drugs and 7,761 adverse events, our approach reveals previously unknown impacts of the pandemic on patient safety and identifies variation of adverse events across patient groups. By disentangling confounders (such as temporal biases in the reporting of adverse events), our approach can detect gender- and age-related variations in adverse events and identify patient groups at higher or lower risk for adverse events during the pandemic relative to time before the pandemic.

Our algorithmic approach led to several key findings. We found substantial variation in adverse drug events before and during the pandemic. Among 64 adverse events identified by our approach, we found that 54 are reported more often during the pandemic, even though adverse event reporting decreased by 4.4% overall. Furthermore, we found that pre-pandemic gender differences are exacerbated during the pandemic. Women suffer from more adverse events than men relative to pre-pandemic levels, across all age groups. We also find relevant clinical differences in adverse event outcomes across age groups. For example, reporting the frequencies of adverse effects such as anxiety and insomnia were disproportionately increased in women and the elderly, indicating they constitute at-risk patient groups. Taken together, these analyses unveil risk-altering adverse events that can inform drug prescription and public health policy, and enable comparison of this pandemic to other health emergencies. Finally, we present a comprehensive catalog of adverse events and their associations. This resource can help discover relationships between drugs and safety events, especially in cases of rare events and effects within population subgroups that differ in their risks of specific clinical outcomes and may be disproportionately affected by preventable inequities.

## Results

### Overview of the approach

We examined 1,425,371 adverse event reports involving 2,821 distinct types of adverse event and spanning 7,761 drugs from the US Food and Drug Administration (FDA) Adverse Event Reporting System (FAERS) dataset, collected between January 2013 and September 2020. The FAERS dataset stores anonymized, manually reviewed adverse event reports received by the FDA. We used the dataset to identify adverse events significantly associated with the pandemic, pinpoint clinically relevant drugs strongly connected with adverse drug events, and identify disparities in the distribution of adverse events across gender and age.

**Fig. 1:**
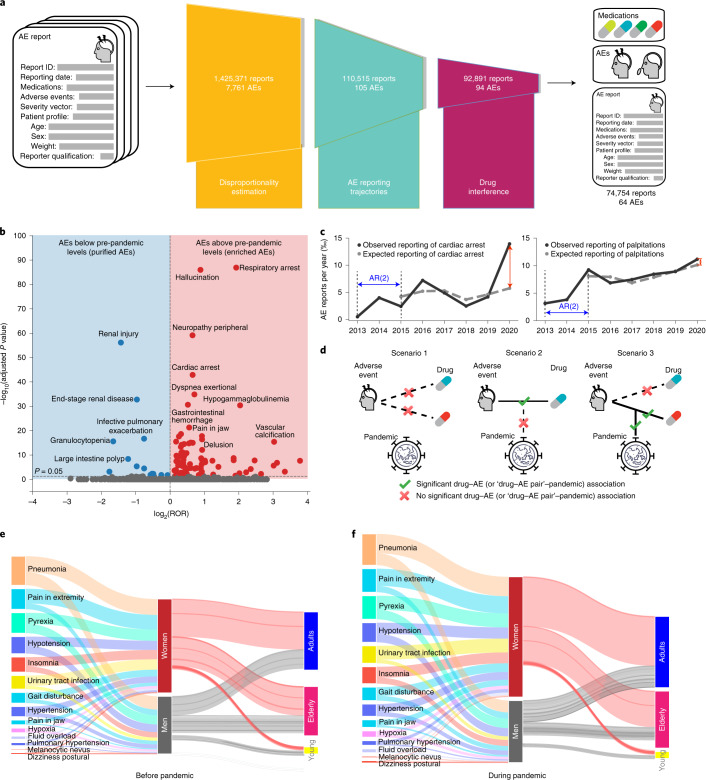
Algorithms for the population-scale analysis of patient drug safety. **a**, Our algorithmic approach detects drug safety signals associated with the pandemic by leveraging a large-scale dataset of adverse event (AE) reports on drugs and their associated adverse reactions. In the overall patient population, it identifies 64 significant types of adverse event, out of 7,761. **b**, Disproportionality estimation. Adverse events with *P* < 0.05 (Bonferroni-corrected) and whose 95% CI of the reporting odds ratio (ROR) does not cross 1 are retained. **c**, Analysis of AE reporting trajectories identifies adverse events with a large gap between the expected and observed reporting. Shown are trajectories of cardiac arrest (left; PAEAI = 1.05; *R*^2^ = 0.49; keep) and palpitations (right; PAEAI = −0.54; *R*^2^ = 0.81; drop). **d**, Drug interference filters adverse events that are not consistently associated with at least one medication and also requires that drug–adverse event pair has a clear association with the pandemic (scenario 3). **e**,**f**, Demographic information before (**e**) and during (**f**) the pandemic. The lengths of bars are proportional to the number of adverse event reports. Shown are adverse drug reactions with PAEAI > 0.8 enriched in women. The total number of reports during the pandemic increased by 28.1% relative to pre-pandemic in women and only by 7.7% in men, implying an increased gender disparity. The difference between lengths of input and output streams in women or men are due to reports with unknown age. AR(2), second-order autoregressive model (Methods). Source data

To this end, we developed an approach that identifies clinically meaningful adverse events that satisfy the following criteria: (1) the reporting frequency of an adverse event changed significantly between 2019 and 2020, (2) the change cannot be explained by its trend in previous years (2013 to 2019) and (3) the adverse drug reaction can be attributed to a specific medication. In line with the three criteria, our approach has three key components (Fig. 1a). First, the approach estimates the reporting odds ratio of every adverse event to identify those whose incidence has shifted considerably during the pandemic (Fig. 1b and Methods, equation (2)). Among these adverse events, the approach then detects those whose change in reporting frequency cannot be explained by the expected upward or downward reporting trajectory had the pandemic not occurred. Existing signal detection methods (such as proportional reporting ratio, reporting odds ratio and Bayesian methods) in pharmacovigilance evaluate the strength of association between a drug and an adverse event^23^ and cannot reliably estimate whether the incidence of an adverse drug reaction is consistent with its temporal trend. By contrast, to quantify deviations from expected trajectories in the reporting frequency during the pandemic, we define the ‘pandemic adverse event association index’ (PAEAI; equation (3) in the Methods). We calculate the PAEAI index for every adverse event and keep those adverse events with positive PAEAI values (Fig. 1c), meaning that their incidence in 2020 substantially deviates from predictions obtained by temporal trend analysis. Finally, our approach identifies adverse events that have considerable associations with specific medications (Fig. 1d; equation (4) in the Methods) while ensuring that the reporting frequencies of the identified drug–adverse event pairs have considerably changed during the pandemic (equation (5) in the Methods).

Taken together, these three components constitute an algorithmic approach that can be used to analyze any patient cohort formed in a population, extract associations between drugs and adverse events, and quantify differential reporting patterns to identify high-risk demographic groups.

### Variation of adverse events before and during the pandemic

We find that most adverse events identified by our approach have increased reporting frequencies during the pandemic, despite a 4.4% decrease in the total number of reports submitted by healthcare professionals, from 220,920 in 2019 to 211,152 reports in 2020. Confirming the validity of our approach, the model detects five adverse events directly related to COVID-19, including coronavirus infection, coronavirus test positive, COVID-19, suspected COVID-19 and COVID-19 pneumonia (all *P* < 10^−37^, two-tailed Fisher’s exact test), which we exclude from the rest of our analyses. The approach detects 64 unique adverse events whose incidence changed during the pandemic in the overall population: 54 have increased reporting frequency during the pandemic and only 10 decreased in frequency (Supplementary Data 1). We refer to adverse drug reactions whose reporting frequency has disproportionately increased with the pandemic onset as ‘enriched adverse events’. Conversely, adverse events with a significant decrease in reporting are referred to as ‘purified adverse events’. Among the 54 enriched adverse events, delusion has the most significant association with the pandemic, with the largest PAEAI score of 1.95. The number of reports involving premature delivery as a drug side effect increased by 73.4%, despite studies from the United States and Europe finding a decrease in the overall incidence of preterm births as a disease during the pandemic^24,25^. Similarly, the incidence of bladder cancers as adverse drug reactions increased by 147%, despite an overall decrease in cancer diagnoses after the onset of the pandemic^9^. The frequency of hallucination side effects increased by 138% during the pandemic, which could be related to reports linking paranoia about COVID-19 to hallucinations as well as long-term neurological impacts of the disease itself^26^. Our approach also detects large increases for severe side effects such as respiratory failure and cardiac arrest. The domination of adverse events with increased frequency is consistently observed in most demographics across sex and age. For example, in patients over 65 years old, 18 out of 19 identified adverse events are enriched and only one is purified (Supplementary Fig. 1).

### Variation of adverse drug events across gender

**Fig. 2:**
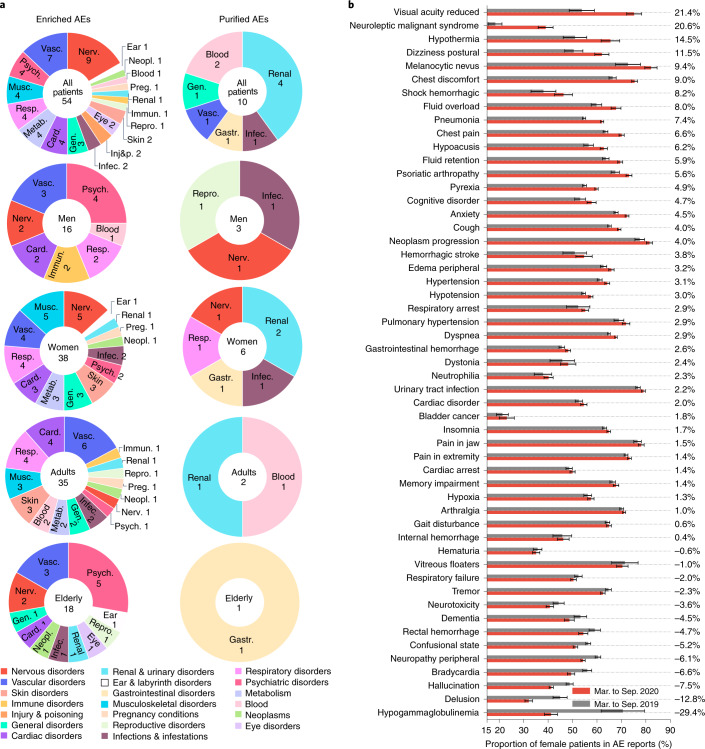
Distribution of identified adverse events. **a**, Identified adverse drug reactions across demographic groups and human organs. Listed are the number of adverse events for every patient group and subdivided into organ systems (Supplementary Section 6). Enriched and purified drug side effects for young patients are shown in Supplementary Figs. 2 and 3. **b**, The proportion of female patients in 53 enriched side effects (omitting reports with unknown sex and excluding premature delivery, which only occurs in women). In the majority (75.5%) of enriched side effects, female patients account for a higher proportion of reports during the pandemic relative to pre-pandemic levels. Standard deviations are calculated via bootstrapping with replacement on patient safety data. Source data

**Fig. 3:**
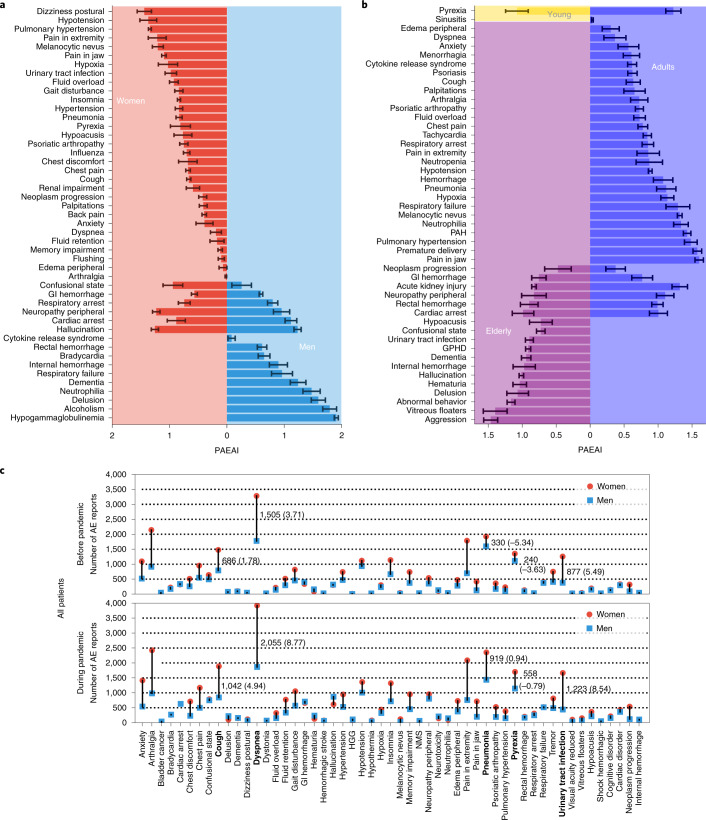
Differential adverse events across gender and age. **a**,**b**, PAEAI differences across gender (**a**) and age (**b**). A higher PAEAI value indicates a more substantial deviation of an adverse event’s incidence from historical trends than expected by simulation. **c**, Gender differences in the reporting of adverse events. Highlighted in bold are five adverse events with the largest differential increase. The first number indicates the absolute difference in the number of reports, and the number in parentheses is normalized by population size (Supplementary Fig. 4). A negative number implies smaller frequency in women than in men. Standard deviations of PAEAIs are calculated via bootstrapping with replacement on patient safety data. GI hemorrhage, gastrointestinal hemorrhage; GPHD, general physical health deterioration; PAH: pulmonary arterial hypertension; HGG, hypogammaglobulinemia; NMS, neuroleptic malignant syndrome. Source data

Although our approach detected 38 adverse events that increased in frequency in women (Supplementary Data 2), only 16 enriched adverse events were detected in men (Supplementary Table 1 and Fig. 2a). This finding is consistent with the over-representation of women in the dataset (62.0% reports from female patients and 38.0% from male patients, excluding reports with unknown sex). Even after accounting for gender differences in the dataset, there are still 48.5 enriched adverse events per million female patients, and only 33.0 adverse events per million male patients. Furthermore, 32 of the 38 side effects are enriched only in women, while only 10 out of 16 are enriched only in men (Fig. 3a). The model identifies six adverse events enriched in both women and men. For example, respiratory arrest has a similar PAEAI in male patients (PAEAI = 0.80) as in female patients (PAEAI = 0.74), suggesting that the pandemic has a comparable influence on the incidence of respiratory arrest in both men and women. In contrast, confusional state has a higher PAEAI in women (PAEAI = 0.94) than in men (PAEAI = 0.26), suggesting that changes brought on by the pandemic exert a greater influence on women.

### Variation of gender proportion in enriched adverse events

We determined the proportion of female patients in each of 54 side effects enriched in the overall population and compared the proportions before and during the pandemic (Fig. 2b). We excluded premature delivery, for which all patients are female. We find that the proportion of female patients increased in 40 out of 53 adverse events during the pandemic. The two adverse events with the largest increases are reduced visual acuity, where the proportion of women increased from 53.8% to 75.2%, and neuroleptic malignant syndrome, where the proportion of female patients increased from 18.4% to 39.0%. We find that the proportion of women in drug-related anxiety reports increased from 67.8% to 72.3%, suggesting that women report anxiety as a side effect at higher rates during the pandemic than men. We observe an increase in the proportion of female reports for side effects associated with anxiety disorders (insomnia, dyspnea (shortness of breath) and dizziness), but a decrease in side effects commonly associated with psychosis disorders (delusion, hallucinations and dementia).

Our analyses identified 13 adverse events for which the proportion of female patients has decreased but the proportion of male patients increased (Supplementary Fig. 7 and Supplementary Section 2). For example, the proportion of hypogammaglobulinemia reports with female patients dropped steeply from 70.6% to 41.2% during the pandemic, despite an eightfold increase in the total number of reports for hypogammaglobulinemia. As patients with hypogammaglobulinemia are immunocompromised and at higher risk for COVID-19, these findings warrant investigation into why the incidence of hypogammaglobulinemia decreased in women (Supplementary Fig. 11), despite an overall increase in reporting and how that relates to undiagnosed cases.

### Variation of adverse drug events across age groups

Stratification of patients by age groups (Fig. 3b) revealed one enriched adverse event in young patients (Supplementary Table 2), 35 in adults (Supplementary Data 3) and 18 in elderly patients (Supplementary Table 3). After accounting for the differences in each patient cohort’s size, there are 68.8 side effects per million adults with increased reporting frequency during the pandemic, compared to 27.8 adverse reactions per million young patients and 58.9 adverse events per million elderly patients. The one side effect enriched in young patients, pyrexia (PAEAI = 1.08), is similarly enriched during the pandemic in adults (PAEAI = 1.22), but is not significantly impacted in the elderly (PAEAI < 0).

Twenty-eight out of the 35 enriched adverse events in adults are unique to adults (not associated with young or elderly patients). For example, drug-related jaw pain has increased incidence in adult patients during the pandemic (PAEAI = 1.61), but not in young or elderly patients. Six adverse drug reactions are enriched in both adult and elderly patients (cardiac arrest, rectal hemorrhage, neuropathy peripheral, acute kidney injury, gastrointestinal hemorrhage and neoplasm progression). Although most have similar PAEAI scores in both cohorts, acute kidney injury has a PAEAI of 1.28 in adults, but 0.81 in elderly patients, suggesting an age-related difference in the pandemic’s impact on drug-related kidney injury. Twelve adverse events are uniquely enriched in elderly patients, including five mental health-related events (hallucination, delusion, aggression, abnormal behavior and dementia). This finding is in contrast with earlier surveys^27^ that showed lower rates of overall anxiety-, depression- and stress-related diseases in elderly compared to younger age groups and suggests that drug-induced psychiatric effects may warrant alternate healthcare interventions.

### Distribution of adverse events across human organs

We grouped 54 adverse events enriched in the overall population into 19 categories based on the System Organ Classification (SOC^28^; Fig. 2a). Nervous and vascular systems events were the most common (Supplementary Fig. 2), with nine and seven adverse events, respectively, suggesting that the incidences of these two adverse event classes are more influenced by the changes brought on by the pandemic. This finding could be related to evidence suggesting COVID-19 can have a considerable impact on the vasculature and can increase the risk for developing neurologic disorders^29^.

Among ten purified adverse events, four are associated with the urinary system while two are blood-related adverse events (Supplementary Fig. 3). Although the incidence decreased in reports submitted by healthcare professionals, we find that five out of ten adverse events (infective pulmonary exacerbation of cystic fibrosis, chronic kidney disease, osteonecrosis of jaw, renal injury and nausea) have more self-reported cases during the pandemic relative to pre-pandemic levels (Supplementary Figs. 8 and 9).

Our approach identified 38 adverse events enriched in female patients and distributed across 14 SOC classes, with the most common classes being nervous, musculoskeletal, vascular and respiratory disorders (Supplementary Figs. 2 and 3). In male patients, 16 enriched adverse events spread across seven SOC classes, with the most common being vascular and psychiatric disorders. Blood and immune system side effects were enriched in men but not women. By contrast, side effects in nine SOC classes that were over-represented in women but not men include metabolism, musculoskeletal, skin, infections and pregnancy-related disorders. Although psychiatric adverse events were enriched in both men and women, there were four psychiatric side effects in men and only two in women—hallucination is enriched in both, anxiety is over-represented in females but not in males, while alcoholism, delusion and dementia are only enriched in male patients.

Psychiatric adverse events were enriched in the elderly, with five enriched adverse events, compared to only one in adults, lending further support to our finding that the elderly may be differentially susceptible to psychiatric side effects of medications. In particular, hallucination, delusion, abnormal behavior, aggression and dementia are enriched in the elderly but not adults, while anxiety is only enriched in adults. Moreover, in the elderly, we observe one eye- and one ear-related adverse event (vitreous floaters and hypoacusis, respectively), but do not detect either in adults. Across sex and age cohorts as well as in the overall population, vascular-related side effects are enriched, suggesting it is a class of adverse drug reactions whose incidence is changed by the pandemic.

### The gender gap in adverse events increased during the pandemic

We investigated gender disparities among the enriched adverse events and whether any pre-existing gender gaps have changed during the pandemic. In the overall population, gender disparities were observed in all 53 adverse events before the pandemic, and we find that those gender differences are exacerbated during the pandemic in 41 out of 53 adverse events (Fig. 3c).

For example, 330 more female patients reported drug-related pneumonia before the pandemic than male patients. During the pandemic, the gap nearly tripled to 919 reports. The gender gaps normalized by population size are shown in Supplementary Figs. 4–6. Similarly, there were 877 more reports of female patients experiencing urinary tract infections (UTIs) than male patients before the pandemic, which is consistent with anatomical and clinical evidence that women are more susceptible to UTIs^30^. The gender gap for this increased to 1,223 reports during the pandemic. In contrast, hallucination showed almost no gender difference (15 cases) before the pandemic. Yet, after the pandemic, male patients reported hallucination more often than women with a large gap of 243 cases. Among the 41 adverse events with an increased gender gap, 33 adverse events involve more female patients than male patients during the pandemic (Fig. 3c).

**Fig. 4:**
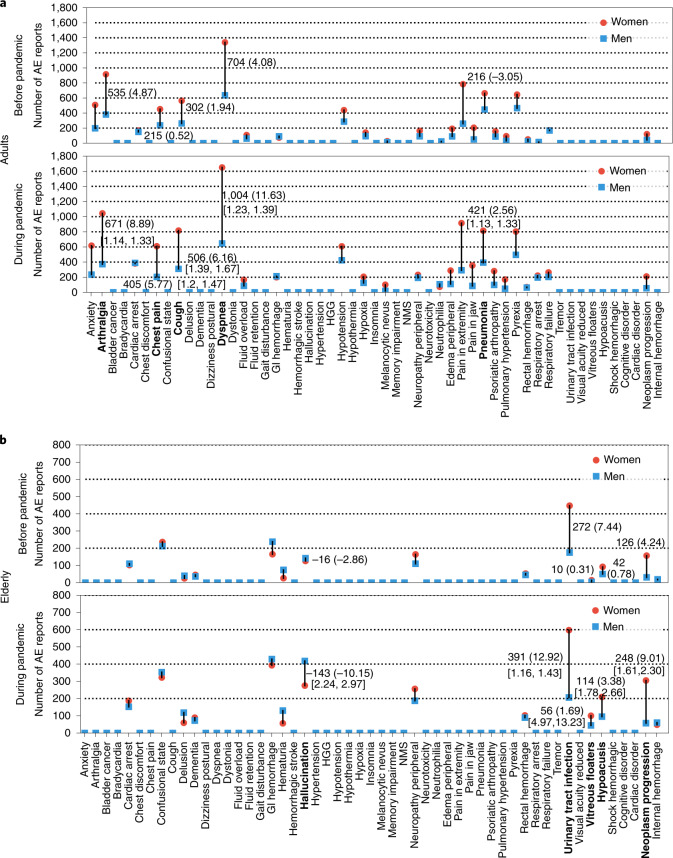
Variation in gender differences across adverse events. **a**, Gender gap in adults. Annotated numbers in square brackets show 95% CI values. Pre-pandemic gender disparities increased in 21 out of 24 types of adverse event during the pandemic. The gender gap (normalized by population size) increased in 17 out of 24 adverse events (Supplementary Fig. 5). **b**, Gender gap in elderly patients. In 13 out of 14 adverse drug events, a pre-existing gender gap has intensified with the pandemic onset. Gender differences increased in 11 out of 14 adverse events during the pandemic (Supplementary Fig. 6; adjusted for population size). Source data

**Fig. 5:**
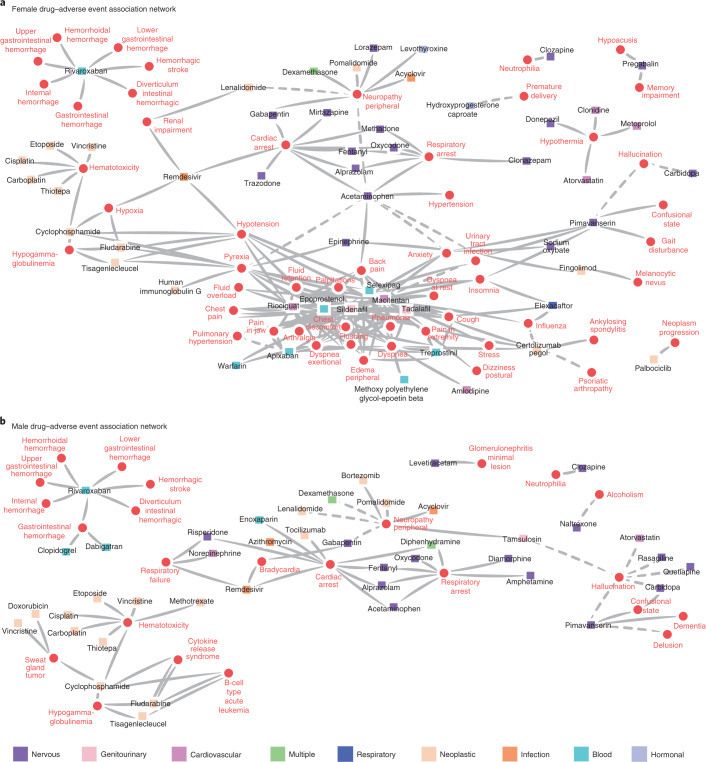
Drug–adverse event associations in gender networks. Networks are constructed from drug–adverse event associations that are disproportionately enriched in women or men during the pandemic. Nodes represent adverse drug reactions (red circles) and drugs (squares). Drugs’ node color is determined by the Anatomical Therapeutic Chemical (ATC) classification system. Only nodes with at least one link are shown. Dashed links indicate that associations may be influenced by disease confounders (Supplementary Data 10 and 11). **a**, Drug–adverse event associations enriched in women. **b**, Drug–adverse event associations enriched in men. Associations between cardiac arrest and nervous system drugs are found in both networks. A link between remdesivir and respiratory failure is found in male but not in female patients.

After stratifying adverse event reports by patients’ age, we observe a similar increase in pre-existing gender differences for adults and the elderly. Among 35 adverse events enriched in adults during the pandemic, 24 have a notable gender difference (10 are only found in reports with unknown sex; excluding premature delivery). Of those, 21 adverse events showed an increased gender gap with onset of the pandemic. Among those 21 drug reactions, 18 involved larger incidence in women than men during the pandemic (Fig. 4a) (Fig. 5 is adjusted for population size). In elderly patients (Fig. 4b; Supplementary Fig. 6 is adjusted for population size), gender differences existed in 14 out of 18 enriched adverse events (four are only observed in reports with unknown sex). The gender differences increased in 13 out of 14 drug reactions during the pandemic. The gender differences in UTIs increased during the pandemic in the elderly but not in adult patients, concordant with evidence that postmenopausal women are most at risk for UTIs as a disease and suggesting they are similarly at risk for drug-related UTIs^30^.

### Gender differences and drug–adverse effect associations

To examine the drug safety landscape across the axis of gender differences, we constructed a network of drug–adverse event associations that were significantly enriched in women (Fig. 5a) and men (Fig. 5b) during the pandemic. Adverse event can be described as either a disease (indication) or a side effect in a report and we accounted for such confounders when calculating drug–adverse event associations (Supplementary Data 10 and 11).

A cluster that includes anxiety and closely associated side effects (such as dizziness) is found only in the female network; one potential reason is the underlying prevalence differences across sexes. For example, women have consistently higher prevalence rates of anxiety induced by medications, which is further exacerbated by the pandemic. The female proportion in patients who have anxiety as an adverse drug reaction is 67.8% before the pandemic and increased to 72.3% during the pandemic (Fig. 2b). Studies on anxiety as an indication found that women are more likely to have reported a mental health disturbance during the pandemic^31^. Our results suggest that the pandemic has also exacerbated the gender gap for anxiety as a drug-related side effect. The comparison of drugs present across both female and male networks is hindered by a lack of information on total drug usage in the dataset. However, two drugs whose on-label use is for erectile dysfunction—sildenafil and tadalafil—are both located within the center of the female-specific cluster. Despite a majority of usage being in men, both drugs are found only in the female cluster. Both have off-label uses for treating sexual dysfunction in women, highlighting our method’s ability to detect potential adverse events of off-label drug usage.

Network topological features of enriched drug–adverse event networks are shared between men and women, such as the cluster of hemotoxicity-associated drugs and the cluster of hemorrhage-related side effects associated with rivaroxaban. In both networks, cardiac and respiratory arrest are associated with a similar cluster of nervous system-related drugs, including common benzodiazapines and opioids such as fentanyl, oxycodone, methadone and diamorphine. Many of these drugs are known to be highly addictive, and this sheds light on how the COVID-19 pandemic has impacted ongoing substance abuse^32^.

We separately analyzed associations with remdesivir as the first treatment for COVID-19 with emergency approval^33^. Remdesivir was present in both male and female networks with an association to cardiac arrest. In our differential analysis of adverse events in patients using remdesivir, we observe that female patients have a higher incidence of hypoxia (Supplementary Section 4), hypotension and renal impairment, while male patients have a higher incidence of respiratory failure. These adverse events have been reported in remdesivir clinical trials^34,35^, but are very rare (cardiac arrest, 1/155 patients; renal impairment, 4/53; hypotension, 4/53; respiratory failure, 2/53)^36^ and the trials fail to provide any differential drug effects for distinct patient subsets. The revealed association with hypoxia (reporting odds ratio = 7.05; 95% confidence interval (CI) = [5.45, 9.12]; *P* < 10^−36^, two-tailed Fisher’s exact test) is not mentioned in the literature. The detection of remdesivir-associated adverse events highlights the importance of population-scale patient safety datasets to detect rare adverse events and unexpectedly high-risk demographic subgroups. As more clinical treatments and vaccines receive emergency use authorization, rapid detection of rare adverse events and stratifying at-risk populations will be critical to patient safety.

## Discussion

Although our approach can effectively identify relationships between adverse events and medications, as well as flag at-risk patient groups during a nationwide pandemic, further research is necessary to pinpoint the driving factors that changed the patient safety landscape so drastically with the onset of the pandemic, including altered drug usage^37^, limited access to healthcare resources^38^ and changes in human behavior (such as less physical activity^39^). Future pharmacoepidemiologic studies can identify informative resources to identify underlying risk factors across gender and age groups^40^. Patient-level data such as healthcare claims^41^ and electronic medical records^42^ can be used to examine the impact of the pandemic on adverse events with finer granularity.

There is an important limitation to consider in interpreting our findings. The patient safety dataset comprises voluntarily submitted reports that are not necessarily representative of the prevalence rates of adverse drug events^43^. However, we mitigate reporting confounders by accounting for two kinds of bias—limited healthcare access and over-reporting due to COVID-19—and show that our results cannot be attributed to those biases (Supplementary Section 3, Supplementary Figs. 10–12, Supplementary Table 4 and Supplementary Data 7). Furthermore, the pandemic has probably affected reporting rates, which can vary across adverse events^44^. The total number of adverse event reports decreased in 2020 relative to 2019; nevertheless, we find that adverse events whose reporting frequencies have changed relative to pre-pandemic levels tend to be reported considerably more often than expected based on historical data. This observation, together with abundant research on clinically relevant insights extracted from patient safety datasets^45,46^, strengthens confidence in our key findings.

Our algorithmic approach can identify differential reporting patterns in patient cohorts formed as a function of gender, age, adverse events and drugs. With additional information on medical and non-medical patient characteristics such as race and ethnicity, this approach is suitable for use in systematic safety surveillance to pinpoint individuals at high risk for safety events based on risk-altering interactions. We also present a comprehensive resource of adverse drug effects and drug–event associations for use in pharmacoepidemiology and public health policy to inform medication use in diverse populations. This resource can guide more focused pharmacological and clinical studies to understand the biological mechanisms and societal impacts of identified adverse event associations. We expect this algorithmic approach to enable comparison of the COVID-19 pandemic to other health emergencies (like the nationwide opioid crisis in the United States and emergencies resulting from hurricanes and wildfires) and to help unveil the disruptive nature public health crises can have on patient safety.

## Methods

The description of the methodology is structured as follows. We start by describing the datasets and their processing and then introduce our algorithmic approach for constructing population-specific models of patient safety.

### Datasets

#### Population-scale patient safety dataset

The adverse event reports used in this work are from the FDA Adverse Event Reporting System (FAERS; https://fis.fda.gov/extensions/FPD-QDE-FAERS/FPD-QDE-FAERS.html), a primary source of post-marketing pharmacovigilance. The reports in FAERS mainly contain demographic information (such as age and sex, but no personal identifiers), drugs (drug substances) and adverse events (preferred terms in MedDRA). We investigated 10,443,476 reports, involving 19,193 adverse events and 3,624 drugs, reported between January 2013 and September 2020. We connected descriptors of adverse events in each report with MedDRA ID preferred terms (Supplementary Section 5) and mapped them to human organ systems through the MedDRA ontology (Supplementary Section 6). Further, we mapped descriptors of drugs to DrugBank IDs and grouped them into categories based on the Anatomical Therapeutic Chemical (ATC) classification system (Supplementary Section 7). For reports with the same case number, we kept only the latest report. We restricted our analysis to adverse events that occurred in the United States (6,351,817 reports) to avoid countrywise biases as well as those biases caused by different national surveillance systems. The World Health Organization (WHO) declared a pandemic on 11 March 2020. At the time of writing, the latest available safety reports were submitted on 30 September 2020. We thus focused on reports submitted from 11 March 2020 to 30 September 2020 and the same period in previous years (from 2013 to 2019), leading to 3,709,531 reports.

#### FDA reporter qualifications

FAERS submissions are voluntarily made by reporters, who send the reports to the FDA directly or through drug manufacturers. The reporters include healthcare professionals (physicians, pharmacists, nurses, dentists and so on) and non-professionals (lawyers and customers). The distribution of reporters was found to be as follows: physicians (594,787, 16.0%), pharmacists (282,323, 7.6%), other healthcare professionals (548,261, 14.8%), lawyers (113,744, 3.1%), customers (2,045,491, 55.1%) and unknown (124,925, 3.37%). Non-healthcare professional reporters lack the domain knowledge to distinguish between true adverse events caused by the drug from indications or unrelated symptoms, or could be over-reporting as a result of panic arising from the pandemic, making these reports more likely to contain false adverse drug reactions that could confound our results. To avoid such confounding, in our main analysis we focused on reports submitted by healthcare professionals (1,425,371 reports). The demographic distribution of the patients is provided in Supplementary Section 8. However, we used all reports submitted by reporters with any qualifications (3,709,531 reports) to investigate the distribution of reporters in some further analyses (Supplementary Figs. 8–10 and 13, Supplementary Table 5 and Supplementary Data 9). Moreover, we compared the results from only using healthcare professional-submitted reports to using all reports (Supplementary Data 8).

### Population-scale adverse event model of patient safety

#### Overview of the approach

Our approach leverages multimodal information—including drugs, adverse events and demographics (for example, gender and age)—from adverse event reports to identify drug side effects that are significantly associated with the pandemic and to detect inequalities among demographic subpopulations. The approach has three components. First, we employ disproportionality estimation to identify adverse drug reactions that are significantly associated with the pandemic. Second, we track the trajectory of each adverse event between 2013 and 2019 and quantify its expected incidence proportion in 2020. We remove any adverse event whose change in reporting frequency during the pandemic can be explained by its temporal trend. Finally, we keep only the adverse events that are significantly associated with at least one drug and where the drug–adverse event pairs are significantly associated with the pandemic.

Next, we take the overall population (Supplementary Data 1) as an example to introduce the pipeline of our proposed framework. Our model can be flexibly generalized to any demographic subpopulation (Supplementary Tables 1–3 and Supplementary Data 2 and 3) by adjusting the input reports. We start with the notation and mathematical representation of the dataset, and then describe details of the three components.

#### Notation and representation of adverse event data

We denote the FAERS dataset as ${{{\mathcal{X}}}}$ where each element **x**_*i*_ represents a single patient safety report. We use ${{{\mathcal{D}}}}$ and ${{{\mathcal{S}}}}$ to denote the set of all drugs and adverse events appearing in the FAERS dataset, respectively. We regard each report as a tuple including a set of drugs ${{{{\mathcal{D}}}}}_{i}$, a set of adverse events ${{{{\mathcal{S}}}}}_{i}$, patient’s age *a*_*i*_, sex *g*_*i*_ and weight *w*_*i*_, reporter’s qualification *q*_*i*_, severity vector **b**_*i*_ and reporting date *t*_*i*_. In other words, we have ${{{{\bf{x}}}}}_{i}=({{{{\mathcal{D}}}}}_{i},{{{{\mathcal{S}}}}}_{i},{a}_{i},{g}_{i},{w}_{i},{q}_{i},{{{{\bf{b}}}}}_{i},{t}_{i})$. The patient may take several medications at the same time and have multiple adverse drug events. Thus, each report contains a drug set ${{{{\mathcal{D}}}}}_{i}$ that is a subset of ${{{\mathcal{D}}}}$, and each drug ${d}_{j}\in {{{{\mathcal{D}}}}}_{i}$ is represented by its DrugBank ID (string). Similarly, the adverse events set ${{{{\mathcal{S}}}}}_{i}\subseteq {{{\mathcal{S}}}}$ contains one or more drug side effects and each ${s}_{h}\in {{{{\mathcal{S}}}}}_{i}$ is represented by its MedDRA ID (string). The patient’s age (in years) *a*_*i*_ is represented by an integer, biological sex is denoted by *g*_*i*_ ∈ {1, 2}, where 1 denotes male and 2 denotes female, and the weight (in kilograms) *w*_*i*_ is represented by a real number. The reporter’s healthcare qualification *q*_*i*_ ∈ {1, 2, 3, 4, 5} falls in one of the five categories of physicians, pharmacists, other professionals, lawyers and customers (denoted by 1 to 5). The severity vector **b**_*i*_ is a binary vector with six elements, where 1 denotes severity and 0 denotes not, corresponding to six outcomes (death, life-threatening condition, hospitalization, disability, congenital anomaly and other medical conditions) of the patient. We represent the reporting date *t*_*i*_ by the number of days between the date when the report is submitted and a defined calibration date (we set it as 1 January 2000). All the introduced components are used for analysis. For instance, we leverage all the available information (such as demographic data, severity and submitting date) for patient matching^47–49^ in the drug interference analysis.

We denote the set of reports submitted in year *k* as ${{{{\mathcal{X}}}}}_{k}$, where *k* ∈ {2013, …, 2020}. The union of every year’s reports is equal to the whole FAERS dataset: $\mathop{\bigcup }\nolimits_{k = 2013}^{2020}{{{{\mathcal{X}}}}}_{k}={{{\mathcal{X}}}}$. In contrast to most traditional post-marketing studies, which only pay attention to specific or few medications and reactions^1^, our research is conducted in a more complex context, involving the time dimension of patient safety data and investigating a large number of drugs and adverse events.

To organize this complex multimodal information we define logical conditions, allowing us to form a cohort of reports as a function of drugs, adverse events and submitting time. A logical predicate *L* consists of a sequence of atomic formulas (drug *d*_*j*_, adverse event *s*_*h*_ and year *k*), which are connected with the following logical connectives: negation (‘not’ or ¬), logical conjunction (‘and’ or ∧), logical disjunction (‘or’ or ∨), existential quantification (∃) and universal quantification (∀). We use ‘⋅’ to denote free/unbound variables. As an example, a logical predicate *L* = (¬*d*_*j*_, *s*_*h*_, ⋅) denotes the following conjunctive connection: ‘Report describes a patient who does not take drug *d*_*j*_ and Report indicates occurrence of adverse event *s*_*h*_ and Report is submitted anytime in 2013–2020 time window’. We define *f*(*L*) as a function of *L* that selects all adverse event reports that satisfy logical predicate *L*. We formulate the value of *f* as

1 $$f({d}_{j},{s}_{h},k)=f(\{{{{\rm{AE}}\,{\rm{report}}}}| L({{{\rm{AE}}\,{\rm{report}}}}({d}_{j},{s}_{h},k))\})=N,$$

where *N* represents the number of adverse event (AE) reports that satisfy the atomic formulas of drug *d*_*j*_, adverse event *s*_*h*_ and submission time *k* connected by logical conjunction. Let us look at an example: *f*(pimavanserin, urinary tract infection, 2020) = 117 selects a set of reports for which the following holds: ‘A patient received pimavanserin treatment and later experienced unwanted side effect of urinary tract infection, and this adverse drug reaction was submitted to the FDA in 2020 (11 March to 30 September).’ In this particular case, there are 117 patients in the FAERS who meet the above requirements.

Our approach uses adverse event data ${{{\mathcal{X}}}}$ and identifies adverse events ${{{\mathcal{S}}}}^{\prime} \subseteq {{{\mathcal{S}}}}$ where each drug side effect ${s}_{h}^{\prime}\in {{{{\mathcal{S}}}}}^{\prime}$ has a significantly different reporting pattern during the pandemic than would have been expected had the pandemic not occurred. To that end, we define three reporting odds ratios (RORs^50,51^): *β*(*s*_*h*_) measures the association between adverse event *s*_*h*_ and the pandemic (Supplementary Data 4); *γ*(*d*_*j*_, *s*_*h*_) quantifies the connection between drug *d*_*j*_ and adverse event *s*_*h*_ (Supplementary Data 5); *δ*(*d*_*j*_, *s*_*h*_) estimates the association between a drug–adverse event pair (*d*_*j*_, *s*_*h*_) and the pandemic (Supplementary Data 6).

#### Step 1 Disproportionality estimation

We conduct the disproportionality estimation^52,53^ on each adverse event to examine the association between the adverse event and pandemic (11 March to 30 September 2020) in contrast to before the pandemic (11 March to 30 September 2019). Although disproportionality analysis is an established approach for pharmacovigilance to generate hypotheses on possible causal relations between drugs and adverse effects^54,55^, we here use it in a different way that quantifies the association between adverse events and their submission periods. For each ${s}_{h}\in {{{\mathcal{S}}}}$, we define *β*(*s*_*h*_) to measure the strength of association between *s*_*h*_ and the pandemic by comparing the reporting frequency during the pandemic with the frequency before the pandemic. Taking *s*_*h*_ as input, we calculate *β*(*s*_*h*_) as

2 $$\beta ({s}_{h})=\frac{f(\cdot ,\,{s}_{h},\,2020)f(\cdot ,\,\neg {s}_{h},\,2019)}{f(\cdot ,\,{s}_{h},\,2019)f(\cdot ,\,\neg {s}_{h},\,2020)},$$

where *f*(⋅, *s*_*h*_, 2020) and *f*(⋅, *s*_*h*_, 2019) represent the number of reports involving *s*_*h*_ in 11 March to 30 September in 2020 and 2019, respectively; *f*(⋅, ¬*s*_*h*_, 2020) and *f*(⋅, ¬*s*_*h*_, 2019) denote the number of reports that do not contain *s*_*h*_ in 11 March to 30 September in 2020 and 2019, respectively. As shown in Supplementary Data 4, we quantify the upper and lower 95% CI of *β*(*s*_*h*_) by ${\rm{e}}^{{\mathrm{ln}}(\beta ({s}_{h}))\pm 1.96\sqrt{1/f(\cdot ,\,{s}_{h},\,2020)+1/f(\cdot ,\,\neg {s}_{h},\,2020)+1/f(\cdot ,\,{s}_{h},\,2019)+1/f(\cdot ,\,\neg {s}_{h},\,2019)}}$. This calculation does not limit the medications listed in the reports.

We calculate significance values using Fisher’s exact test followed by the Bonferroni correction for multiple hypothesis testing. We keep only adverse events that pass both the significance test (adjusted *P* < 0.05) and the ROR criterion. For *β*(*s*_*h*_), the ROR-based selection criterion is that the lower 95% CI is greater than 1 for adverse events that are reported more frequently during the pandemic (enriched or over-represented), or that the upper 95% CI is less than 1 for adverse events that are reported less often during the pandemic (purified or under-represented). Unlike previous studies that mainly focused on drug responses with higher ROR (such as ROR > 1)^20^, our model can detect both enriched (*β*(*s*_*h*_) > 1) and purified (*β*(*s*_*h*_) < 1) adverse events.

#### Step 2 Adverse event reporting trajectories

The reporting trajectory of an adverse event refers to the changing trend of adverse event incidence proportion, indicated by its temporal/historical data. For example, if the reporting frequency of a certain adverse drug reaction has continually increased from 2013 to 2019, it would be expected to also grow from 2019 to 2020, and we cannot attribute its increased incidence to the pandemic. We develop the PAEAI index to measure whether an adverse event’s incidence conforms to its predicted trajectory.

We regard 11 March to 30 September in 2020 as the pandemic period and the same interval in previous years (2013 to 2019) as the non-pandemic periods. For *s*_*h*_ that pass the twofold criterion in the previous step, we build a trajectory vector **v**(*s*_*h*_) = [*v*_*h*,2013_, *v*_*h*,2014_, …, *v*_*h*,2020_]. Element *v*_*h*,*k*_ represents the proportion of reports related to *s*_*h*_ in all reports submitted in year *k*. For example, of the 211,152 reports submitted during the pandemic period, 6,130 involve hallucination, which means the proportion of hallucination during the pandemic is 2.9% = 6,130/211,152. Inspired by the powerful temporal feature capture ability of autoregressive methods^56^, we train a second-order autoregressive regression (AR(2); Supplementary Figs. 14 and 15) model for each adverse event by fitting its historical values [*v*_*h*,2013_, *v*_*h*,2014_, …, *v*_*h*,2019_]. The regression models trained on different adverse events do not share parameters. The optimized model is then used to predict the proportion of *s*_*h*_ in each year. The predictions $[{v}_{h,2015}^{\prime},\,{v}_{h,2016}^{\prime},\ldots ,\,{v}_{h,2020}^{\prime}]$ are from 2015 to 2020 since the two-order AR model needs the first two data points (in 2013 and 2014) as initial inputs. On top of the difference between observation and prediction, we define the PAEAI of *s*_*h*_ as

3 $${{{\rm{PAEAI}}}}({s}_{h})={{\mathrm{log}}}\,\frac{| {{{{\bf{r}}}}}_{2020}({s}_{h})| }{\frac{1}{5}\mathop{\sum }\nolimits_{k = 2015}^{2019}| {{{{\bf{r}}}}}_{k}({s}_{h})| },$$

where **r**_*k*_(*s*_*h*_) denotes the standardized residual in year *k* in the regression model of *s*_*h*_, which is calculated through ${{{{\bf{r}}}}}_{k}({s}_{h})={{{{\bf{e}}}}}_{k}({s}_{h})/\sqrt{\left({\frac{1}{6}}\right)\mathop{\sum }\nolimits_{k = 2015}^{2020}{{{{\bf{e}}}}}_{k}^{2}({s}_{h})}$. The ${{{{\bf{e}}}}}_{k}({s}_{h})={v}_{h,k}^{\prime}-{v}_{h,k}$ denotes the residual of *s*_*h*_ in year *k* (from 2015 to 2020).

In summary, the PAEAI index measures the ratio of the average standardized residual during the pandemic relative to the non-pandemic period (Supplementary Section 1). A positive PAEAI indicates the reporting frequency of *s*_*h*_ has changed during the pandemic more than would have been expected had the pandemic not occurred. It suggests that the change in the reporting frequency of adverse event *s*_*h*_ is associated with the pandemic and cannot be explained by temporal trends based on historic data. A negative PAEAI indicates that the change during the pandemic does not exceed expected normal fluctuations of the *s*_*h*_’s trajectory. A higher value of PAEAI indicates more substantial changes in the reporting frequency of *s*_*h*_. As PAEAI uses the logarithm function, a small difference in PAEAI reflects a rather large change during the pandemic relative to pre-pandemic levels. In the next step of the approach we only consider *s*_*h*_ with positive PAEAI.

#### Step 3 Drug interference

Next, we reduce the confounding effects of multiple drug associations. Traditional pharmacosurveillance typically focuses on how adverse events are connected to a specific medication^57^. However, our approach is able to discover multiple drugs that may explain a change in reporting frequency during the pandemic. We consider two types of interference from drugs:

- The adverse event co-occurs with a certain drug but their association may not be significant.
- The association between an adverse event and the pandemic can be attributed to multiple drugs; however, none of the formed drug–adverse event pairs are significantly associated with the pandemic.

Accordingly, we consider two criteria to prevent drug interference. First, the adverse event (such as a urinary tract infection) should be significantly associated with the therapy of at least one drug (like pimavanserin). Second, the formed drug–adverse event pair (such as pimavanserin–urinary tract infection) should be significantly associated with the pandemic.

To eliminate the first type of interference, for a certain adverse event *s*_*h*_ that has passed the selection in the first two steps of our approach, we go through all the drugs that co-occurred with it in adverse event reports during the pandemic (that is, ${{{{\mathcal{X}}}}}_{2020}$). We use ${{{{\mathcal{N}}}}}_{{s}_{h}}$ to denote the set of found drugs for *s*_*h*_. We only check associations between *s*_*h*_ and ${d}_{j}\in {{{{\mathcal{N}}}}}_{{s}_{h}}$ during the pandemic (for simplification, we omit the subscript of the year). For a specific drug ${d}_{j}\in {{{{\mathcal{N}}}}}_{{s}_{h}}$, we regard the reports where the drug is involved as positive samples (${{{{\mathcal{X}}}}}_{{d}_{j}}$), and the remaining reports as negative samples (${{{{\mathcal{X}}}}}_{\neg {d}_{j}}$). All positive samples are included in test group ${{{{\mathcal{T}}}}}_{{d}_{j}}$. To keep the comparison fair, we select a subset from negative samples as control group ${{{{\mathcal{C}}}}}_{{d}_{j}}$ where the patients are similar to the ones from ${{{{\mathcal{T}}}}}_{{d}_{j}}$ (that is, ${{{{\mathcal{T}}}}}_{{d}_{j}}={{{{\mathcal{X}}}}}_{{d}_{j}}$ and ${{{{\mathcal{C}}}}}_{{d}_{j}}\in {{{{\mathcal{X}}}}}_{\neg {d}_{j}}$)^52,58^. The reports in ${{{{\mathcal{C}}}}}_{{d}_{j}}$ are selected through a nearest-neighbor propensity score matching model^58^, which measures the similarity between a report (a patient) from negative samples with a report from positive samples as a function of the patient characteristics. Based on the available information, we build a characteristic factor **z**_*i*_ = [*a*_*i*_, *g*_*i*_, *w*_*i*_, *q*_*i*_, *b*_*i*,1_, …, *b*_*i*,6_, *t*_*i*_] for each report, including the patient’s age, sex, weight, the qualification of the reporter, severity vector (*b*_*i*,1_ to *b*_*i*,6_ are the six elements in **b**_*i*_) and the submission date. For each report in test group ${{{{\mathcal{C}}}}}_{{d}_{j}}$, we select 10 reports that have the highest propensity scores into the control group^52^, which makes $| {{{{\mathcal{C}}}}}_{{d}_{j}}| =10| {{{{\mathcal{T}}}}}_{{d}_{j}}|$. The propensity scores are measured by the cosine similarity among characteristic factors. Afterwards, based on ${{{{\mathcal{C}}}}}_{{d}_{j}}$ and ${{{{\mathcal{T}}}}}_{{d}_{j}}$, we define *γ*(*d*_*j*_, *s*_*h*_) for each *s*_*h*_ and all its co-occurring drugs ${d}_{j}\in {{{{\mathcal{N}}}}}_{{s}_{h}}$ by

4 $$\gamma ({d}_{j},\,{s}_{h})=\frac{f({d}_{j},\,{s}_{h},\,2020)f(\neg {d}_{j},\,\neg {s}_{h},\,2020)}{f({d}_{j},\,\neg {s}_{h},\,2020)f(\neg {d}_{j},\,{s}_{h},\,2020)},$$

where *f*(*d*_*j*_, *s*_*h*_, 2020) represents the quantity of reports with a certain drug *d*_*j*_ and the specific drug reaction *s*_*h*_; *f*(*d*_*j*_, ¬*s*_*h*_, 2020) is the number of reports with *d*_*j*_ but not *s*_*h*_; *f*(¬*d*_*j*_, *s*_*h*_, 2020) is the number of reports with *s*_*h*_ but not *d*_*j*_; *f*(¬*d*_*j*_, ¬*s*_*h*_, 2020) is the number of reports without *d*_*j*_ and without *s*_*h*_. We quantify the upper and lower 95% CI of *γ*(*d*_*j*_, *s*_*h*_) using ${\rm{e}}^{{\rm{ln}}(\gamma ({d}_{j},\,{s}_{h}))\pm 1.96\sqrt{1/f({d}_{j},\,{s}_{h},\,2020)+1/f({d}_{j},\,\neg {s}_{h},\,2020)+1/f(\neg {d}_{j},\,{s}_{h},\,2020)+1/f(\neg {d}_{j},\,\neg {s}_{h},\,2020)}}$ (Supplementary Data 5).

We set the criteria for significance to be that the 95% CI of *γ*(*d*_*j*_, *s*_*h*_) does not cross 1 and the *P* value corrected by the Bonferroni method is smaller than 0.05. To this end, for each *s*_*h*_ we have a drug set ${{{{\mathcal{N}}}}}_{{s}_{h}}^{\prime}$ that is a subset of ${{{{\mathcal{N}}}}}_{{s}_{h}}$, where each drug ${d}_{j}\in {{{{\mathcal{N}}}}}_{{s}_{h}}^{\prime}$ is significantly associated with *s*_*h*_.

To address the second type of interference from drug associations, we assess whether each drug–adverse event pair is significantly associated with the pandemic. We only consider the *s*_*h*_ that are significantly associated with at least one drug (that is, ${{{{\mathcal{N}}}}}_{{s}_{h}}^{\prime}\ne \varnothing$). We then define *δ*(*d*_*j*_, *s*_*h*_) as denoting the odds ratio of pair (*d*_*j*_, *s*_*h*_) (where ${d}_{j}\in {{{{\mathcal{N}}}}}_{{s}_{h}}^{\prime}$) during the pandemic:

5 $$\delta ({d}_{j},\,{s}_{h})=\frac{f({d}_{j},\,{s}_{h},\,2020)f(\neg {d}_{j},\,\neg {s}_{h},\,2019)}{f({d}_{j},\,{s}_{h},\,2019)f(\neg {d}_{j},\,\neg {s}_{h},\,2020)},$$

where *f*(*d*_*j*_, *s*_*h*_, 2020) and *f*(*d*_*j*_, *s*_*h*_, 2019) denote the number of reports that contain the drug–adverse event pair of interest in 2020 and 2019, respectively; *f*(¬*d*_*j*_, ¬*s*_*h*_, 2020) and *f*(¬*d*_*j*_, ¬*s*_*h*_, 2019) denote the number of reports that do not contain the (*d*_*j*_, *s*_*h*_) pair in 2020 and 2019, respectively. Through ${\rm{e}}^{{\rm{ln}}(\delta ({d}_{j},\,{s}_{h}))\pm 1.96\sqrt{1/f({d}_{j},\,{s}_{h},\,2020)+1/f(\neg {d}_{j},\,\neg {s}_{h},\,2020)+1/f({d}_{j},\,{s}_{h},\,2019)+1/f(\neg {d}_{j},\,\neg {s}_{h},\,2019)}}$, we calculate the upper/lower 95% CI of *δ*(*d*_*j*_, *s*_*h*_) (Supplementary Data 6). The criterion of significance is the same as the previous stage (that is, 95% CI of *δ*(*d*_*j*_, *s*_*h*_) does not cross 1 and Bonferroni-adjusted *P* < 0.05). Thus, for *s*_*h*_, we have a set of drugs, denoted by ${\widehat{{{{\mathcal{N}}}}}}_{{s}_{h}}$ and ${\widehat{{{{\mathcal{N}}}}}}_{{s}_{h}}\subseteq {{{{\mathcal{N}}}}}_{{s}_{h}}^{\prime}$, where each drug ${d}_{j}\in {\widehat{{{{\mathcal{N}}}}}}_{{s}_{h}}$ satisfies the criterion that the drug–adverse event pair (*d*_*j*_, *s*_*h*_) is significantly associated with the pandemic. Any adverse event ${s}_{h}^{\prime}$ with ${\widehat{{{{\mathcal{N}}}}}}_{{s}_{h}}\ne \varnothing$ satisfies the criterion that its change in reporting frequency can be attributed to at least one medication, and the drug–adverse event pair is significantly associated with the pandemic.

In summary, each adverse event ${s}_{h}^{\prime}$ identified by our approach has multiple defined metrics. The *β* (along with 95% CI and adjusted *P* value) detects the reporting frequency during the pandemic, which is above (over-represented) or under (under-represented) what we expected (‘Step 1 Disproportionality estimation’). The PAEAI describes whether the change in reporting frequency in 2020 cannot be explained by the adverse event’s temporal trend from 2013 to 2019 (‘Step 2 Adverse event reporting trajectories’). Each ${s}_{h}^{\prime}$ has one or more *γ* and *δ* (and corresponding 95% CIs and adjusted *P* values) to ensure that the change to reporting frequency during the pandemic is not affected by drug interference.

#### Statistics and reproducibility

Hypothesis testing is carried out using two-tailed Fisher’s exact test. Bonferroni correction is used to correct for multiple hypothesis testing. We calculate the ROR to measure the effect size and report upper and lower 95% confidence intervals (CIs). We reject a null hypothesis when the adjusted *P* value is smaller than 0.05 and the 95% CI range does not cross 1. We use bootstrapping to estimate error bars in the PAEAI index calculation. We ensure the reproducibility of our analysis by clearly presenting the proposed method and provide publicly accessible code and data.

### Supplementary information

Supplementary Information Supplementary sections 1–8, Figs. 1–15 and Tables 1–5.

Supplementary Data 1 Summary of identified adverse events across all patient groups. Shown are 64 adverse events (54 enriched and 10 purified events) identified in the overall population by the proposed method.

Supplementary Data 2 Summary of identified adverse events in female patients. Shown are 44 adverse events, including 38 enriched and 6 purified events during the pandemic, identified in female patients.

Supplementary Data 3 Summary of identified adverse events in adult patients. Shown are 37 adverse events, including 35 enriched and 2 purified events, identified in adults.

Supplementary Data 4 Associations between adverse events and the pandemic.

Supplementary Data 5 Associations between adverse events and drugs.

Supplementary Data 6 Associations between drug–adverse event pairs and the pandemic.

Supplementary Data 7 Popularity-based rank of adverse events. Shown are the top 50 adverse events most frequently reported in COVID-19 publications during the pandemic.

Supplementary Data 8 Results comparison using different report sources. Summary of analysis results while using reports from healthcare professionals and from all reporters, respectively.

Supplementary Data 9 Distribution of reporters for each enriched adverse event. Shown are the number of reports submitted by category of qualification for each of 54 adverse events that are enriched in all patients.

Supplementary Data 10 Summary of statistics for adverse events overlaps with indications in female patients. Shown are statistics of reports for drug–adverse event pairs identified in women.

Supplementary Data 11 Summary of statistics for adverse event overlaps with indications in male patients. Shown are statistics of reports for drug–adverse event pairs identified in men.

### Source data

Source Data Fig. 1 Results of disproportionality estimation and historical trend; demographic distribution before and during the pandemic.

Source Data Fig. 2 Variations of female proportion in identified adverse events.

Source Data Fig. 3 PAEAI across sex and age; variation of gender gap in all patients.

Source Data Fig. 4 Variation of gender gap in adults and elderly patients.

## Data Availability

All data used in this paper, including the raw and processed adverse event report dataset, adverse event ontology, drug ontology and the results of our analyses, are shared with the research community via the project website at https://zitniklab.hms.harvard.edu/projects/patient-safety. The raw adverse event reports are obtained from the FDA Adverse Event Reporting System (FAERS): https://fis.fda.gov/extensions/FPD-QDE-FAERS/FPD-QDE-FAERS.html. The raw adverse event ontology data from MedDRA are available at https://www.meddra.org/, which requires subscription. The raw drug mapping data from DrugBank are available at https://go.drugbank.com/releases/latest. All data supporting the findings of this study are also available in Harvard Dataverse repository^59^: 10.7910/DVN/G9SHDA. All Supplementary data (1–11) are available with this manuscript. Source data are provided with this paper.

## Code availability

Python implementation of the methodology developed and used in the study is available at https://zitniklab.hms.harvard.edu/projects/patient-safety. The code to reproduce all results, documentation and examples of usage, are available at figshare^60^ (10.6084/m9.figshare.15088161.v1) and also on GitHub (https://github.com/mims-harvard/patient-safety).
