# Supplementary material for: Population-scale identification of differential adverse events before and during a pandemic
Source: Nat Comput Sci. 2021 Oct 5;1(10):666–77. doi: 10.1038/s43588-021-00138-4 (PMC10766557; doi:10.1038/s43588-021-00138-4)
Supplement: Supplementary file 1 — Supplementary sections 1–8, Figs. 1–15 and Tables 1–5. [file 43588_2021_138_MOESM1_ESM.pdf]

nature portfolio
https://doi.org/10.1038/s43588-021-00138-4

# Supplementary information

# Population-scale identification of differential adverse events before and during a pandemic

In the format provided by the
authors and unedited

Supplementary Information for

# Population-scale identification of differential adverse events before and during a nationwide pandemic

Xiang Zhang[1], Marissa Sumathipala[1], and Marinka Zitnik[1,2,3,‡]

[1]Department of Biomedical Informatics, Harvard University, Boston, MA 02115, USA

[2]Broad Institute of MIT and Harvard, Cambridge, MA 02142, USA

[3]Harvard Data Science Initiative, Cambridge, MA 02138, USA

‡Corresponding author. Email: marinka@hms.harvard.edu

This PDF file includes:

Supplementary Sections 1 to 8

Supplementary Figures 1 to 15

Supplementary Tables 1 to 5

## List of Supplementary Sections


1 Quantification of the results of AE reporting trajectories . . . . . . . . . . . . . . . . . S4
2 Additional information on proportion of male patients . . . . . . . . . . . . . . . . . S4
3 Possible confounding of detected associations with the willingness of individuals to report adverse events . . . . . . . . . . . . . . . . . . . . . . . . . . . . . . . . . S4
4 Further details on the association between Remdesivir and hypoxia . . . . . . . . . . S6
5 Adverse event identification . . . . . . . . . . . . . . . . . . . . . . . . . . . . . S6
6 Mapping of adverse events to human organ systems . . . . . . . . . . . . . . . . . . S7
7 Mapping of drugs, controlled drug vocabulary, and Anatomical Therapeutic Chemical (ATC) classification system . . . . . . . . . . . . . . . . . . . . . . . . . . . . S7
8 Demographic distribution in dataset . . . . . . . . . . . . . . . . . . . . . . . . . S7


## List of Supplementary Figures


1 Number of identified adverse events across demographic groups. . . . . . . . . . . . S8
2 Distribution of enriched adverse events by SOC class and demographic group. . . . . S9
3 Distribution of purified adverse events by SOC class and demographic group. . . . . S10
4 Gender gap in all patients normalized by population size. . . . . . . . . . . . . . . S11
5 Gender gap in adults normalized by population size. . . . . . . . . . . . . . . . . . S12
6 Gender gap in elderly normalized by population size. . . . . . . . . . . . . . . . . S13
7 Comparison of proportion of male patients in enriched adverse events before and during the pandemic. . . . . . . . . . . . . . . . . . . . . . . . . . . . . . . . . S14
8 Comparison of reporter distribution in purified adverse events before and during the pandemic (continued in Supplementary Figure 9). . . . . . . . . . . . . . . . . . . . S15
9 Comparison of reporter distribution of purified adverse events before and during the pandemic (continued). . . . . . . . . . . . . . . . . . . . . . . . . . . . . . . . . S16
10 Comparison of the total number of adverse event reports before and during the pandemic.S17
11 Publication counts for adverse events. . . . . . . . . . . . . . . . . . . . . . . . . S18
12 Ranks of COVID-related symptoms. . . . . . . . . . . . . . . . . . . . . . . . . . S19
13 Comparison of reporter distribution of hypogammaglobulinemia before and during the pandemic. . . . . . . . . . . . . . . . . . . . . . . . . . . . . . . . . . . . . S20
14 Model fitting comparison between autoregressive models trained on observed data and randomly permuted data. . . . . . . . . . . . . . . . . . . . . . . . . . . . . . S21
15 Prediction results comparison between autoregressive models trained on observed data and randomly permuted data. . . . . . . . . . . . . . . . . . . . . . . . . . . S22


## List of Supplementary Tables


1 Summary of adverse events identified by our approach in male patients. . . . . . . . S23
2 Summary of adverse events identified by our approach in young patients. . . . . . . . S24
3 Summary of adverse events identified by our approach in elderly patients. . . . . . . S24

4 Summary of COVID-related symptoms. . . . . . . . . . . . . . . . . . . . . . . . . . . S25
5 Summary statistics of dataset. . . . . . . . . . . . . . . . . . . . . . . . . . . . . . . . S26

# Supplementary Sections

## 1 Quantification of the results of AE reporting trajectories

In our study, we evaluate the reporting trajectories of 105 adverse events, including both overrepresented and underrepresented ones, whose reporting frequencies are significantly associated with the pandemic based on estimations from upstream analysis. There are 94 out of 105 (89.5%) adverse events with positive PAEAI, indicating their reporting frequency during the pandemic violates their trends prior to 2020: these adverse events may point to patient safety issues and require our attention. In contrast, our model detects 11 out of 105 adverse events (10.5%) with negative PAEAI and follow their trajectories: their change in reporting frequency during the pandemic can be attributed to historical trends.

## 2 Additional information on proportion of male patients

Of the 54 enriched adverse events, we find 13 adverse events for which male patients have a higher proportion during the pandemic period compared to the same period in 2019 (Supplementary Figure 7). Among these, our model identifies two side effects with changes exceeding 10%: hypogammaglobulinemia and delusion with increase of 29.4% and 12.8%, respectively. For example, the proportion of males among all patients who reported delusion as a drug side effect, submitted by professional health workers, is 55.1% in 2019 but rapidly increased to 67.9% during the pandemic: warranting more attention to the safety of men's medications.

## 3 Possible confounding of detected associations with the willingness of individuals to report adverse events

The detected adverse events that have higher reporting frequencies during the pandemic could result from two factors: 1) more individuals suffer from adverse drug reactions during the pandemic; 2) the people experiencing drug side effects is at the same level as pre-pandemic but a larger proportion of them report to the FDA. In order to disentangle these two effects and remove the confounder of varying reporting ratios, we consider two types of biases associated with reporting rates and address how our key findings cannot be attributed to these biases.

**Bias caused by limited access to professional medical resources.** During the pandemic, public medical resources are dominantly occupied by COVID-related patients and most people avoid visiting healthcare facilities (such as hospitals) to minimize risk of transmission [1]. Therefore, even if customers have a higher willingness to report adverse drug reactions during the pandemic, they may be more likely report by themselves (i.e., self-reporting) or through their insurance lawyers: these reporters are regarded as non-professional reporters in FAERS. To validate this assumption, we analyze the distribution of reporters (Supplementary Figure 10). Results show that reports submitted by non-healthcare professionals increased by 13.8% during the pandemic period relative to the same period before the pandemic, in contrast, those submitted by healthcare providers decreased by 4.4% To disentangle the bias presented by limited healthcare access, we analyze reports from healthcare

professionals separately from the reports submitted by consumers and other non-healthcare professionals.

**Bias caused by people who have access to professional healthcare workers.** Concern and panic relating to COVID-19 may inflate the reporting rate, even if the true incidence of an adverse drug reaction is unaffected by the pandemic. To address this confounding factor:

- We consider the number of publications in the NCBI PubMed database (https://www.ncbi.nlm.nih.gov/pubmed) as a proxy measure that allows us to systematically and objectively determine how much attention each adverse event draws during the pandemic. In particular, we use the NCBI Entrez Programming Utilities (https://biopython.readthedocs.io/en/latest/chapter_entrez.html) to retrieve the number of available publications in PubMed (before September 30, 2020) for each of 7,761 adverse events. The original 19,193 adverse events are narrowed down to 7,761 after limiting by country, reporter's qualification, submitting period, and SOC. We count one publication if its abstract contains both the adverse drug reaction and "COVID" (or "SARS-CoV-2"). We rank the adverse events by their popularity (i.e., the number of publications) as shown in Supplementary Figure 11 and report the top 50 popular adverse drug reactions in Supplementary Data 7. We also rank the adverse events by the ROR (calculated in the first step of our model) and evaluate the relationship between popularity-based ranking and ROR-based ranking. We found that the Spearman rank-order correlation coefficient is close to zero (coefficient = -0.007; p-value = 0.521). A not significant result means that the popularity of an adverse event is not significantly related to the results detected by our model, indicating the detected adverse events are not confounded by the attention they drawn from society.

- We next regard the symptoms of COVID-19 as another proxy measure to investigate the confounding factor that patients over-report symptoms as side effects to their healthcare providers. We have retrieved 17 common symptoms associated with COVID-19 from the US CDC (https://www.cdc.gov/coronavirus/2019-ncov/symptoms-testing/symptoms.html) and WHO (https://www.who.int/emergencies/diseases/novel-coronavirus-2019/advice-for-public): dyspnoea (i.e., difficulty breathing), cough, pyrexia (i.e., fever), pneumonia, nausea, chest pain, rash, vomiting, headache, diarrhoea, respiratory symptom, respiratory tract infection, cardiovascular disorder, skin disorder, respiratory disorder, lung disorder, and dermatosis. For these symptoms, we find significant differences between their ROR-based ranks and popularity-based ranks (Supplementary Figure 12; Student's T-test, p-value $< 10^{-24}$), indicating that our model is confounded by the increased popularity of COVID-19 symptoms. We report the symptoms in the Supplementary Table 4. Moreover, only a small proportion of the 54 adverse events our model detected as enriched during the pandemic (five symptoms: dyspnoea, cough, pyrexia, pneumonia, and chest pain) overlap with the set of COVID-19 symptoms. Furthermore, one of the COVID-19 symptoms (nausea) is even identified as purified by our method (Supplementary Table 4; the reporters' distribution of nausea is shown in Supplementary Figure 9). The above evaluations

together consistently support that our findings are not confounded by the increased attention given to COVID-19 symptoms.

If the enriched adverse events detected by our model are confounded by the public's reporting intention, we would expect to see those that are tightly associated with the pandemic to be largely overrepresented. However, the above analyses show that our model does not simply detect the most popular adverse drug reactions (nor symptoms) during the pandemic and our findings can not be explained by changes to people's willingness to report adverse events.

## 4 Further details on the association between Remdesivir and hypoxia

As hypoxia may be an indication for Remdesivir [2], it is important to disentangle this possible confounding factor for the association between Remdesivir and hypoxia that our model detected. Among the 3,019 reports involving Remdesivir, 98 reports include hypoxia as a side effect, 2 reports list hypoxia as both indication and side effect, and 10 reports list it as an indication but not a side effect. The remaining 2,909 reports do not include hypoxia (*i.e.*, neither reported hypoxia as a disease nor adverse event). We calculate the association between two variables that 'hypoxia is reported as side effect of Remdesivir' and 'hypoxia is reported as indication of Remdesivir' through odds ratio. The p-value of a Fisher's exact test is larger than 0.05, indicating it is not related that hypoxia as a disease and as a side effect in Remdesivir-treated patients. In other words, our finding that hypoxia is a side effect of Remdesivir is not confounded by indications.

## 5 Adverse event identification

The drug reactions recorded in FAERS reports are characterized by preferred terms (PT; text string) in MedDRA ontology (https://www.meddra.org/how-to-use/basics/hierarchy) [3]. We map the adverse events from their PT string in FAERS to the corresponding MedDRA PT identifier for downstream analysis. One of the major challenges in mapping is that the string names (*e.g.*, 'Phobia fear \\ Height') shown in adverse event reports may not match with the standard MedDRA PT names because of nonstandard annotations. To address this issue, we propose a simple but efficient mapping strategy which contains three steps. First, we convert all strings into lower case (*e.g.*, 'phobia fear \\ height'); second, split the string by '\\' if exist, and then separately map the split strings (*e.g.*, 'phobia fear' and 'height') to MedDRA; third, if none of them match with the standard PT names, we further split the string by '␣' if exist (*e.g.*, 'phobia' and 'fear') and check the matching (*e.g.*, 'phobia' match with MedDRA ID '10034912'). In this way, we can map 98.2% of all the adverse events strings appeared in FAERS dataset, which is higher than most studies in literature (such as [4]). We analyze separately reports containing explicit references to COVID-19. That included 6 adverse events: COVID-19 (1,674 reports), COVID-19 pneumonia (135 reports), suspected (103 reports), asymptomatic (14 reports), coronavirus infection (547 reports), and coronavirus test positive (103 reports). When analyzing gender differences, we treat sex-specific adverse drug reactions separately. For example, when measuring the impact of the pandemic on gender gaps, we manually excluded premature

delivery because it only occurs in female patients.

## 6 Mapping of adverse events to human organ systems

To explore the disparities among broader classes of adverse events, we categorize adverse events, from the 'Preferred Terms (PTs)' level to the 'System Organ Classes (SOCs)' level, based on etiology (such as infections and infestations) and manifestation sites (such as gastrointestinal disorders) following the MedDRA hierarchy. We manually remove adverse events in four SOCs (*i.e.*, social circumstances, surgical and medical procedures, product issues, and investigations) that are not related to medications. Furthermore, to increase the robustness and generalizability of our results, we focus on the adverse events that are observed in at least 100 reports either before or during the pandemic.

## 7 Mapping of drugs, controlled drug vocabulary, and Anatomical Therapeutic Chemical (ATC) classification system

We first map the drugs in the AE reports to DrugBank IDs (https://go.drugbank.com) [5]. We implement the same mapping strategy as described in the section on mapping of adverse events. We also group drugs into categories given by the Anatomical Therapeutic Chemical (ATC) classification system. The ATC categorization is an internationally accepted classification system maintained by the WHO (https://www.whocc.no/atc_ddd_index) [6] that classifies active ingredients of drugs according to the organ or system on which they act and their therapeutic, pharmacological and chemical properties. For example, drug Ritonavir is annotated with ATC codes J05AR10, J05AP53, J05AE03, J05AR23, J05AR26, and J05AP52, indicating that Ritonavir is an antiviral drug used for treatment of HIV and HCV infections.

## 8 Demographic distribution in dataset

The proportion of men, women, and unknown sex are 484,649 (34.0%), 784,230 (55.0%), and 156,492 (11.0%), respectively. Based on the aging criteria set by the WHO [7], we split the patients into young ($<$20 years, 35,987 reports, 2.52% of all reports, mean=13.9, std=9.0), adults (20$-$65 years, 508,983 reports, 35.7%, mean=47.8, std=13.2), elderly ($>$65 years, 305,685, 21.4%, mean=72.9, std=11.1), and unknown age (574,716 reports, 44.3%). In sex- or age-specific analysis, we omit reports with unknown sex or unknown age, respectively. For instance, we ignore patient reports with unknown sex when calculating the proportion of female patients in adverse event reports (Figure 2b).

## Supplementary Figures

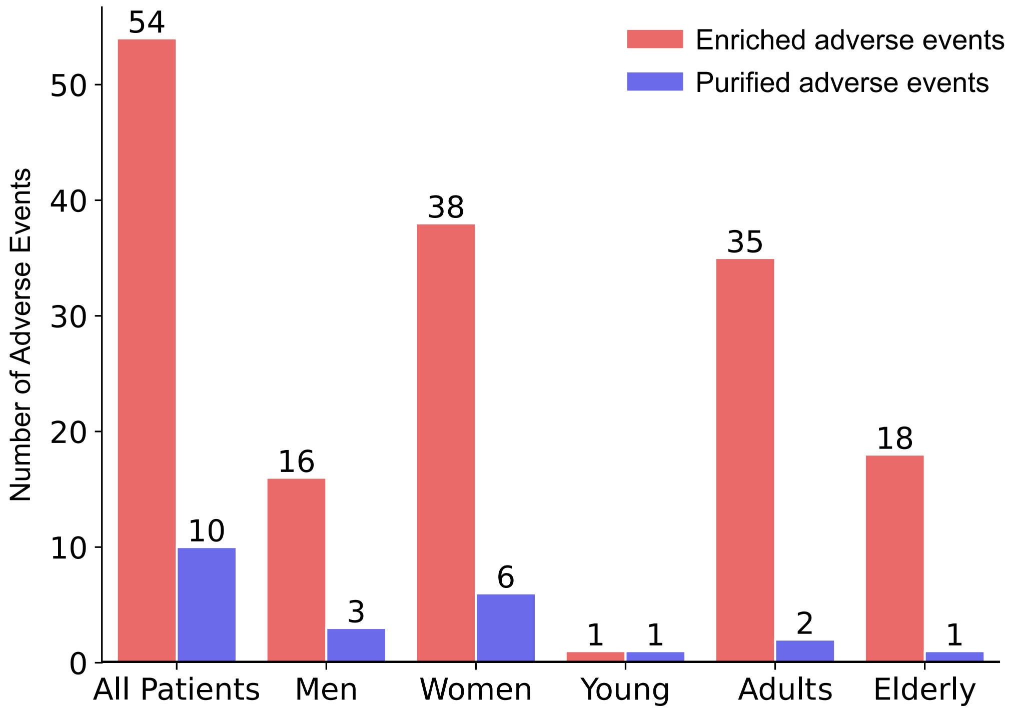


**Supplementary Figure 1: Number of identified adverse events across demographic groups.** Enriched adverse events refer to the drug side effects that are reported more often during the pandemic compared to before the pandemic, while purified ones are reported less frequently. Although the total amount of reports submitted in the pandemic period (March 11 – September 30, 2020) has dropped to 211,152 in contrast to 220,920 before the pandemic (March 11 – September 30, 2019), we find that the enriched adverse drug reactions are more than, if not equal to, the purified ones. In terms of all patients, the number of enriched adverse events is 5.4 times more than the purified adverse events. This difference is consistently observed across demographic groups including men (5.33×), women (6.33×), adults (17.5×), and elderly (18×). We find female patients suffer from more enriched adverse events than male patients and adults more than young and aged individuals. After adjusted by the size of the patient populations in our dataset, we find 48.5 adverse events (per million patients) enriched in women, compared to only 33.0 in men. There are only 27.8 (per million patients) enriched adverse drug reactions in young people, compared to 68.8 in adults and 58.9 in elderly patients.

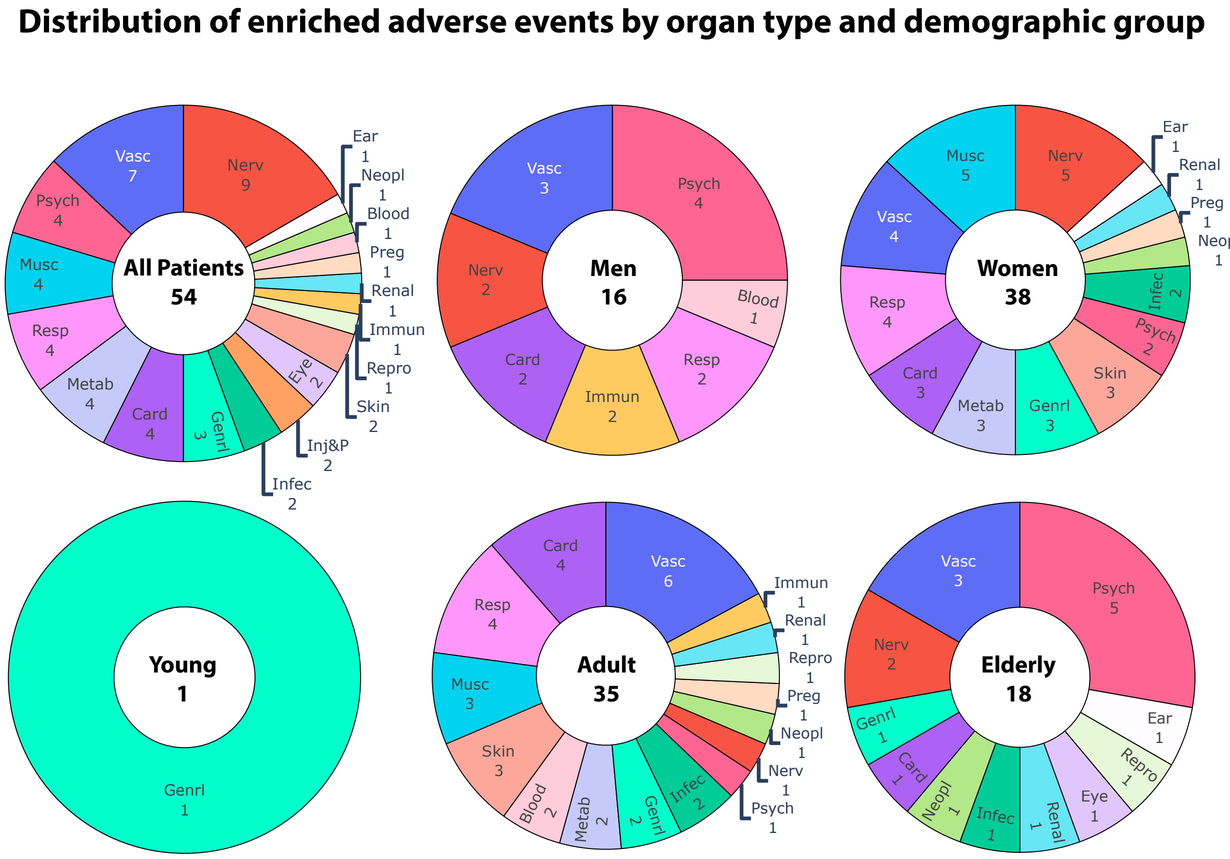


**Supplementary Figure 2: Distribution of enriched adverse events by SOC class and demographic groups.** The colors represent organ classes (based on System Organ Classification (SOC) in the MedDRA hierarchy; Methods) and the legend is the same as in Figure 2a. We find some SOC classes are over-represented among the enriched adverse events for different patient populations. For example, several psychology-related side effects are enriched in male patients but not in female patients. Our model also detects the different classes of enriched adverse events in diverse patient cohorts, compared to the overall population. There are nine adverse events related to the nervous system that are enriched in the overall population, compared to only one in adults and two in elderly patients.

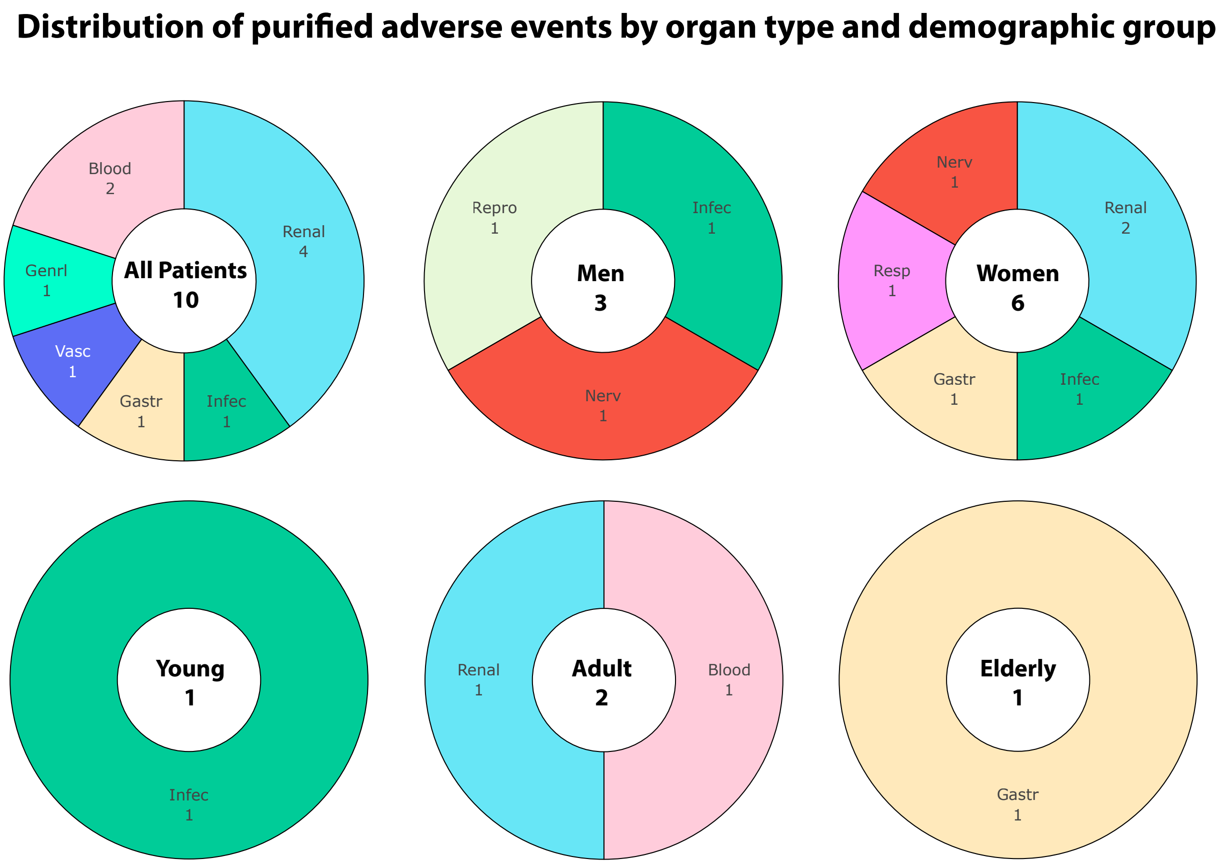


**Supplementary Figure 3: Distribution of purified adverse events by SOC class and demographic groups.** The legend is the same as in Figure 2a where color represents organ systems according to the system organ class (SOC) in MedDRA ontology. This figure presents the adverse events, identified by our framework, reported less frequently during the pandemic (only considering the reports submitted by healthcare professionals (Methods)). For example, we find 4 out of 10 purified adverse events in all patients are related to the renal system, which could be because diagnosis of renal adverse events depends on access to professional medical facilities that are restricted during the pandemic. To further take into account the cases reported by nonprofessionals, we investigate the distribution of reporters' qualification of each purified adverse event (Supplementary Figure 8; Supplementary Figure 9).

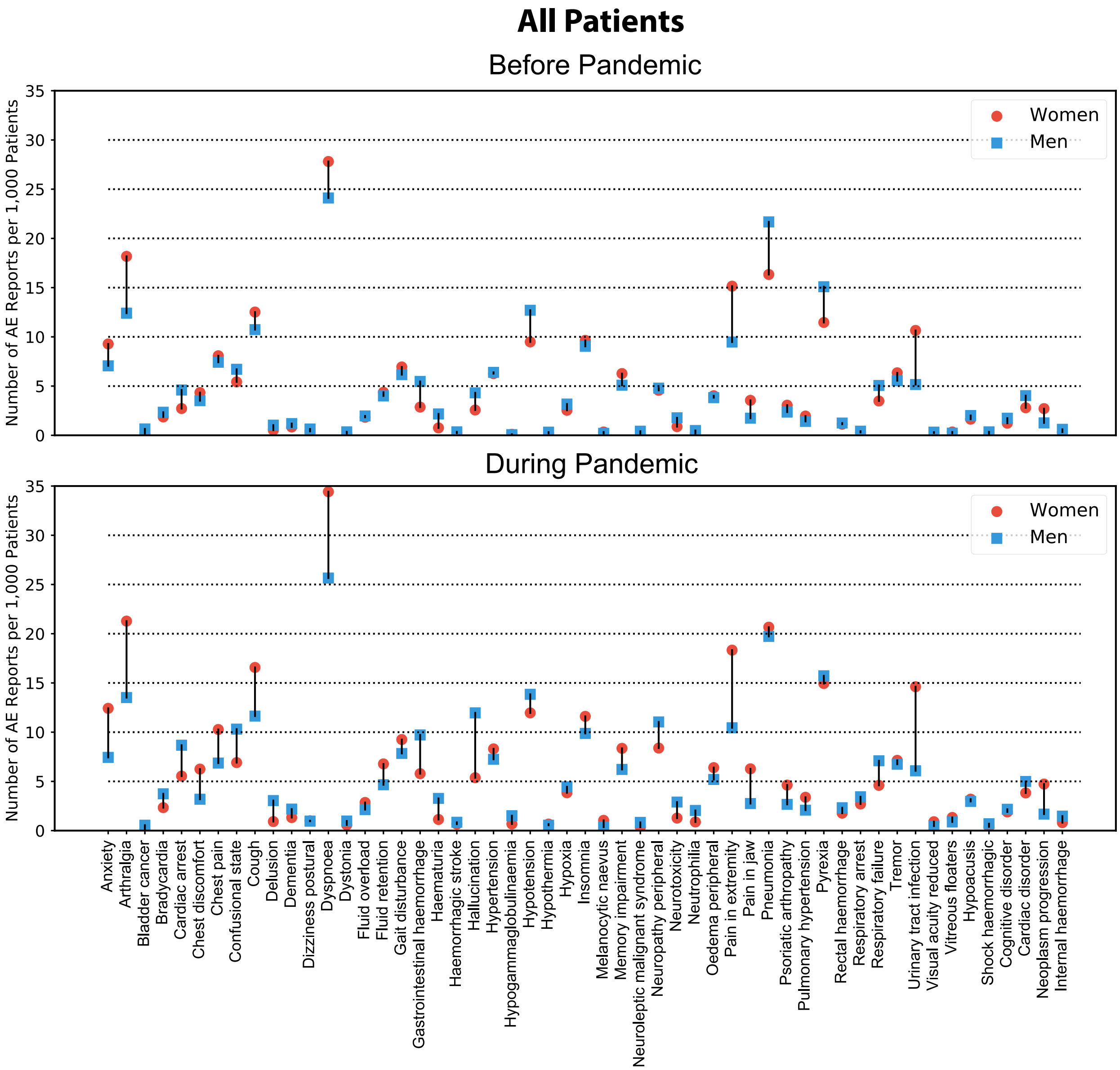


**Supplementary Figure 4: Gender gap in all patients normalized by population size.** We provide the incidence proportion (*i.e.*, the number of reports regarding a specific adverse event in 1,000 patients) of the enriched adverse events. This figure corresponds to Figure 3c (in the main text) whose y-axis shows the absolute number of reports. We find that gender differences are increased in most adverse events (62.3%) during the pandemic compared to before the pandemic.

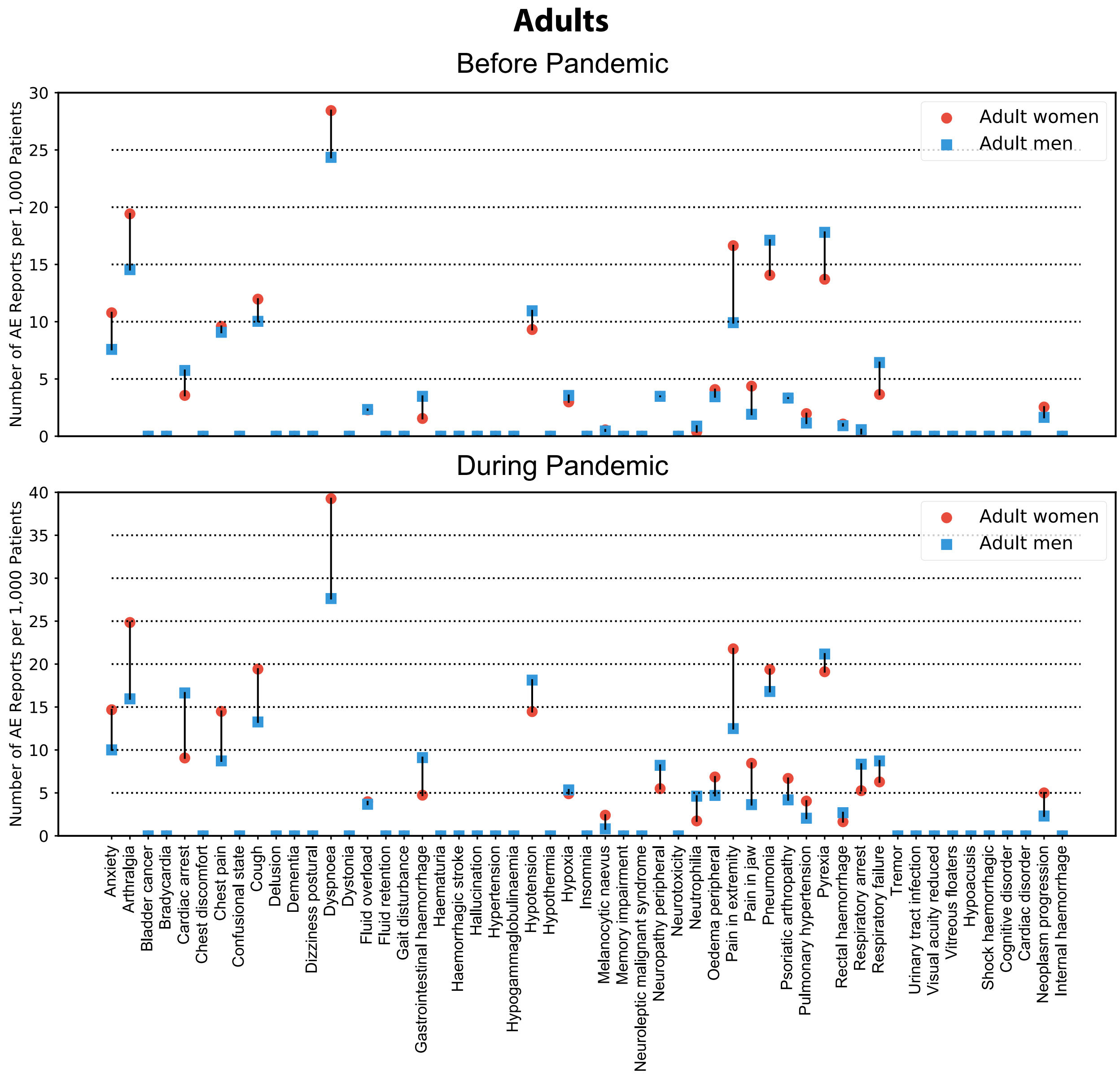


**Supplementary Figure 5: Gender gap in adults normalized by population size.** This figure corresponds with Figure 4a that shows the absolute number of reports. The y-axis in this figure presents the relative number of reports adjusted by the number of adult patients (before and during the pandemic, respectively). For better comparison with the counterparts in all patients, we provide all adverse events as shown in the Supplementary Figure 4. The adverse events with zero reports per thousand adults are not significantly associated with the pandemic in adults (although significant in the overall population), or are only observed in unknown sex. Among 24 drug reactions with gender disparities, we observe that 17 have a larger gender gap during the pandemic.

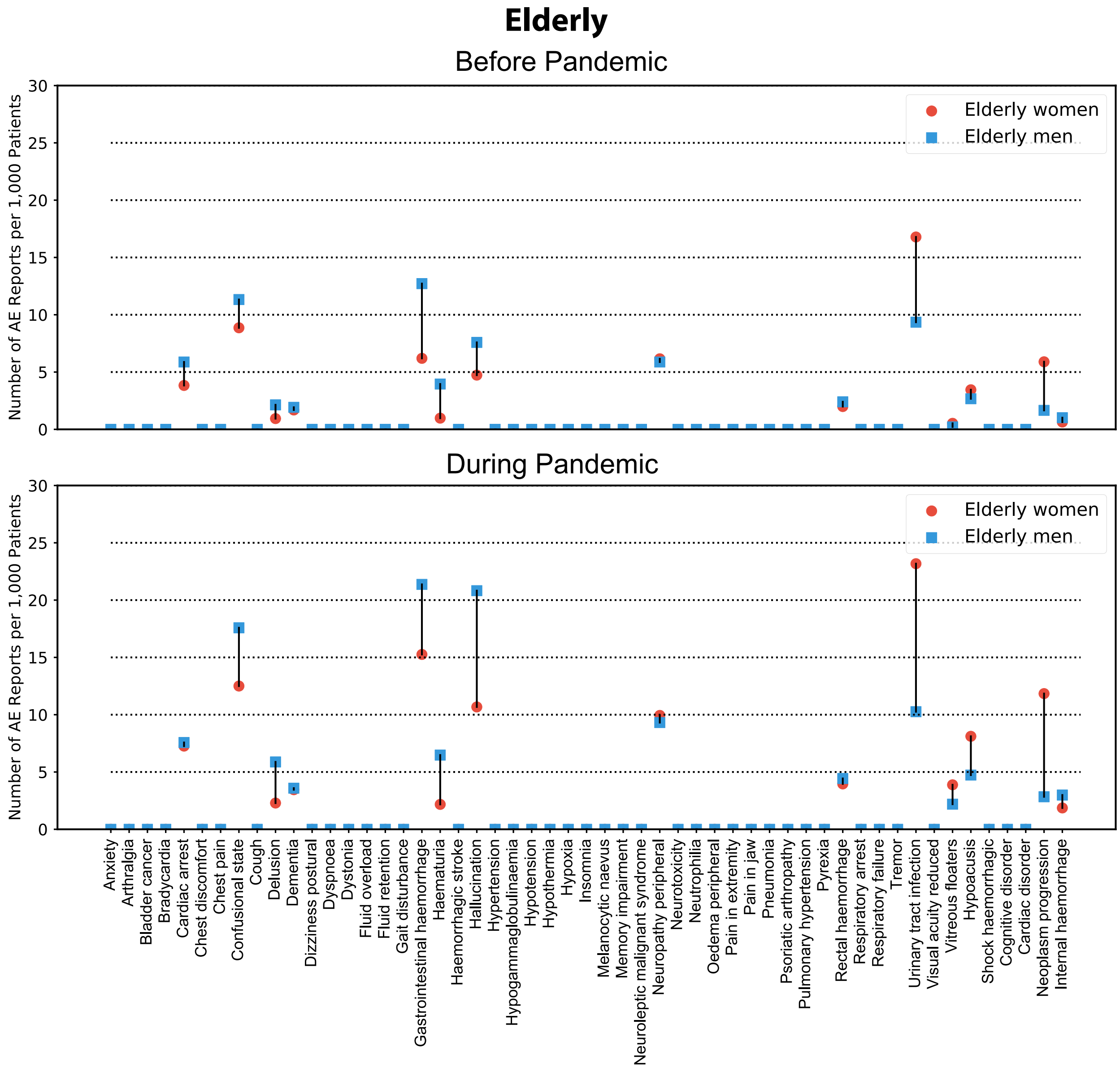


**Supplementary Figure 6: Gender gap in elderly normalized by population size.** This figure corresponds to Figure 4b with different y-axis (the y-axis in this figure presents the relative number of reports adjusted by the number of elderly patients before and during the pandemic, respectively). For better comparison with the adverse events identified in the overall patient population, we provide all adverse events as shown in Supplementary Figure 4. The adverse events with zero reports per thousand patients are not significantly associated with the pandemic in elderly (although significant in the overall patient population), or are only observed in unknown sex. We find most (78.6%) of pre-existing gender gaps are increased during the pandemic. For example, before the pandemic, there are 11.33 reports involving confusional state in each thousand female patients, which is 2.47 more than its counterpart in male patients (8.86); the gender difference grows to 5.08 during the pandemic (17.58 in men; 12.5 in women).

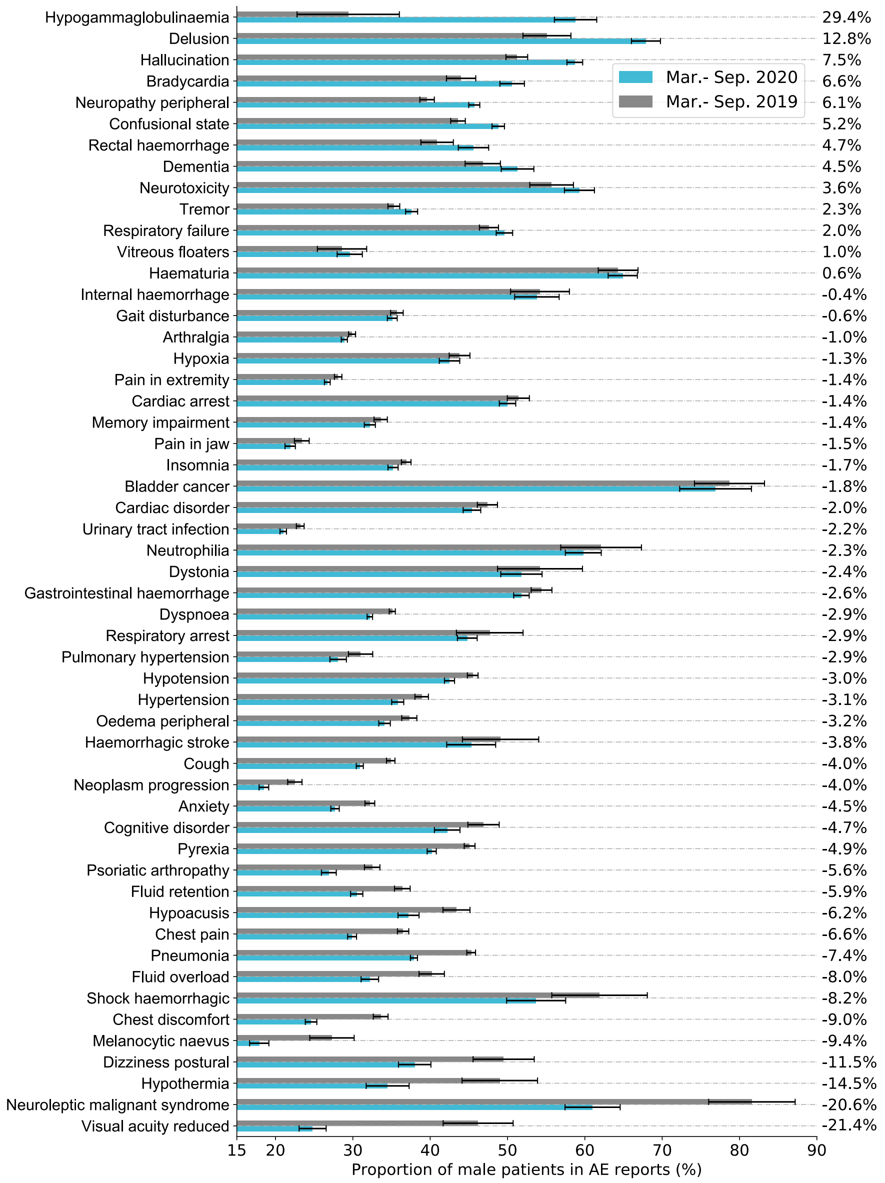


**Supplementary Figure 7: Comparison of proportion of male patients in enriched adverse events before and during the pandemic.** We present the percentage of men (omit unknown sex) in adverse event reports, analogous to Figure 2b which shows the proportion of female patients in adverse event reports. The annotation shows the increased magnitude during the pandemic (March 11 – September 30, 2020) compared to before the pandemic (March 11 – September 30, 2019). We find the proportion of male patients are increased in 13 out of 53 enriched adverse events during pandemic period. For example, among the reports containing hypogammaglobulinaemia as a drug side effect, only 29.5% occurred in men before the pandemic but jumped to 58.9% after the pandemic onset.

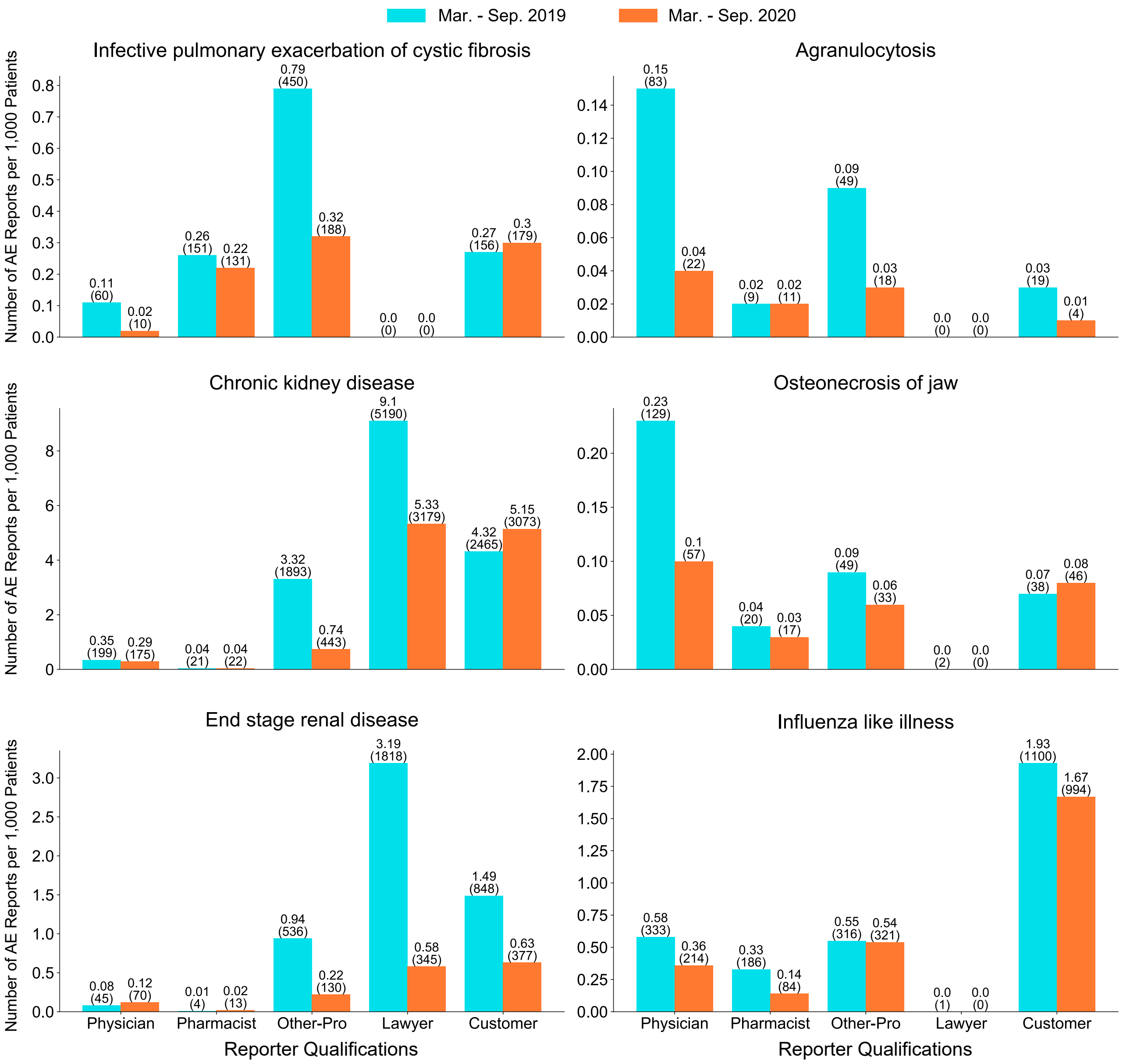


**Supplementary Figure 8: Comparison of reporter distribution in purified adverse events before and during the pandemic (continued in Supplementary Figure 9).** The adverse event reports in FAERS are submitted by five categories of reporters: physician, pharmacist, other professionals (such as nurses), lawyer, and customer. Based on the reports submitted by healthcare professionals (the first three qualifications), our approach identifies 10 purified adverse events in all populations. Here, we provide fine-grained reporter distribution by extending to nonprofessional reporters (the last two qualifications). The y-axis shows the number of reports per 1,000 patients and the annotations in brackets present the absolute number of reports.

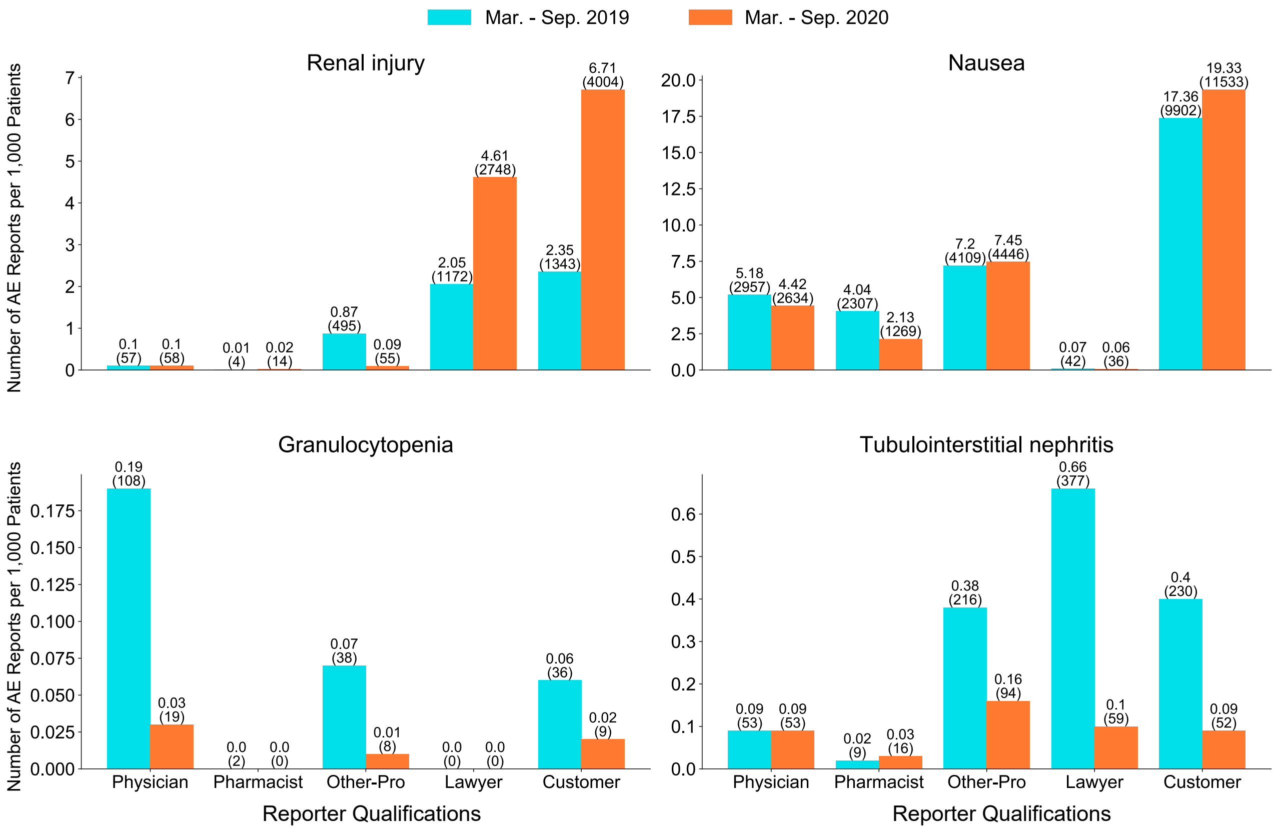


**Supplementary Figure 9: Comparison of reporter distribution of purified adverse events before and during the pandemic (continued).** Although the incidence proportions are decreased in reports submitted by professionals for all 10 purified adverse events, we find five adverse events (infective pulmonary exacerbation of cystic fibrosis, chronic kidney disease, osteonecrosis of jaw, renal injury, and nausea) have an increase in self-reported cases (submitted by customer themselves) during the pandemic. In particular, reports of kidney injury have dropped dramatically among professionals, but have risen sharply among non-professionals, which suggests that patients suffering from drug related kidney injury may not have sufficient professional medical resources during the pandemic.

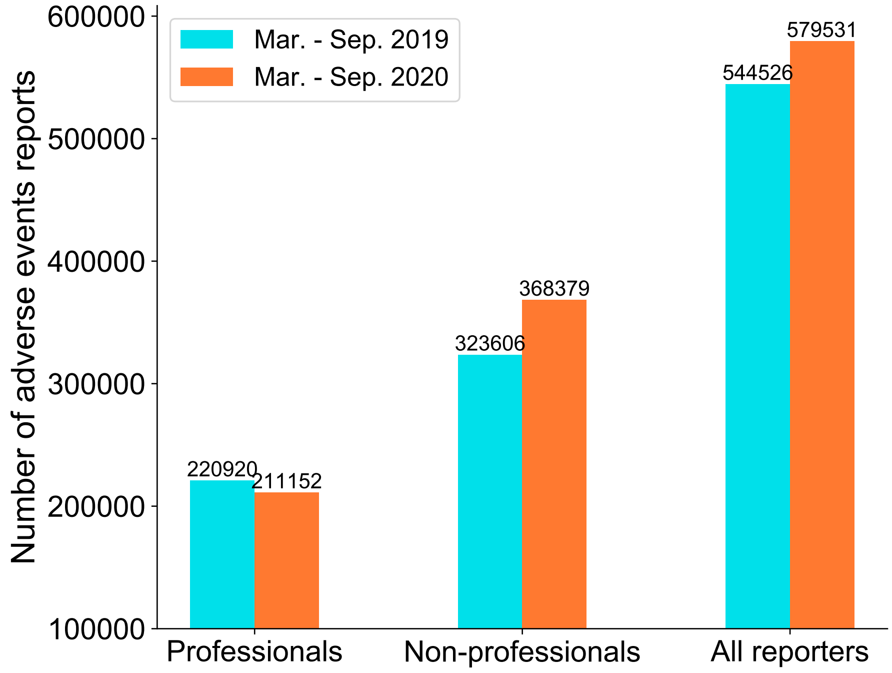


**Supplementary Figure 10: Comparison of the total number of adverse event reports before and during the pandemic.** During the pandemic period (March 11-September 30, 2020), compared to the same period before the pandemic (March 11-September 30, 2019), the number of adverse events reports submitted by all reporters has increased 6.4%. However, the reports from healthcare professional reporters dropped by 4.4% while those from non-professionals increased by 13.8%. One potential reason is that individuals are more likely to report their experiences about adverse drug reactions during the pandemic but do not have the ability to report to healthcare professionals due to limited access to healthcare during the pandemic.

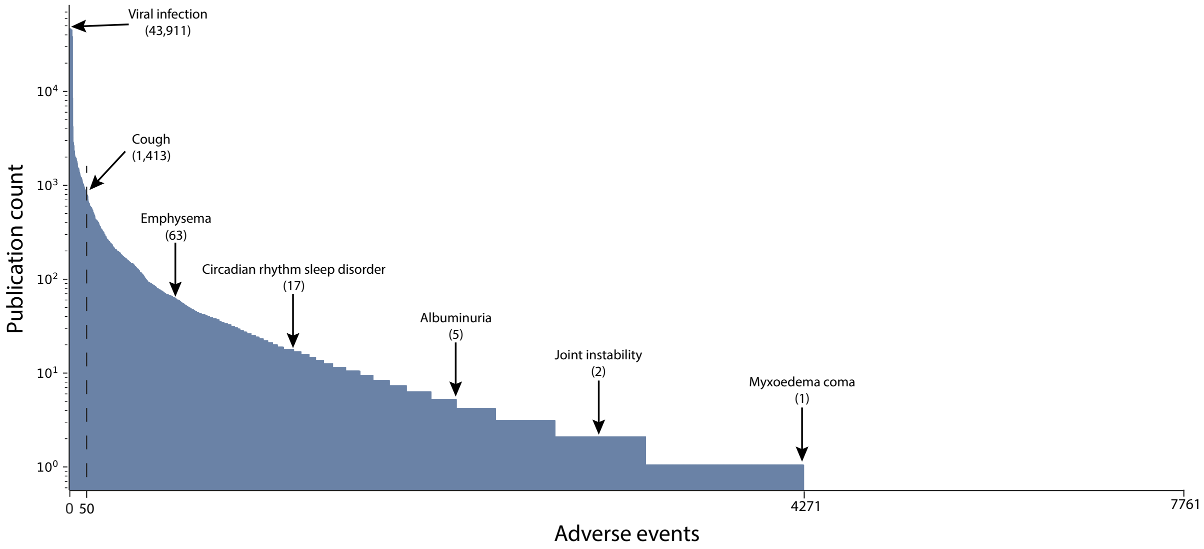


**Supplementary Figure 11: Publication counts for adverse events.** The number of publications in the NCBI PubMed database (https://www.ncbi.nlm.nih.gov/pubmed) for each of 7,761 adverse events. In particular, we use the NCBI Entrez Programming Utilities (https://biopython.readthedocs.io/en/latest/chapter_entrez.html) to retrieve the number of available publications in PubMed before September 30, 2020. We count one publication if its abstract contains both the adverse drug reaction and 'COVID' (or 'SARS-CoV-2'). We rank the adverse events by their popularity (i.e., the number of publications) and report the top 50 popular adverse drug reactions (prior to the dashed vertical line) in Supplementary Data 7. We find 4,271 adverse events co-occurred with 'COVID' (or 'SARS-CoV-2') in publications, with the most popular one being a viral infection (appeared 43,911 times). We rank the adverse events by the ROR calculated in the first step of our model (Methods) and evaluate the relationship between popularity-based ranking and ROR-based ranking. We found that the Spearman rank-order correlation coefficient is close to zero (coefficient $\rho$ = -0.007; p-value = 0.521. A not significant result means popularity/attention is not significantly related to the results detected by our model, based on a non-parametric permutation test.

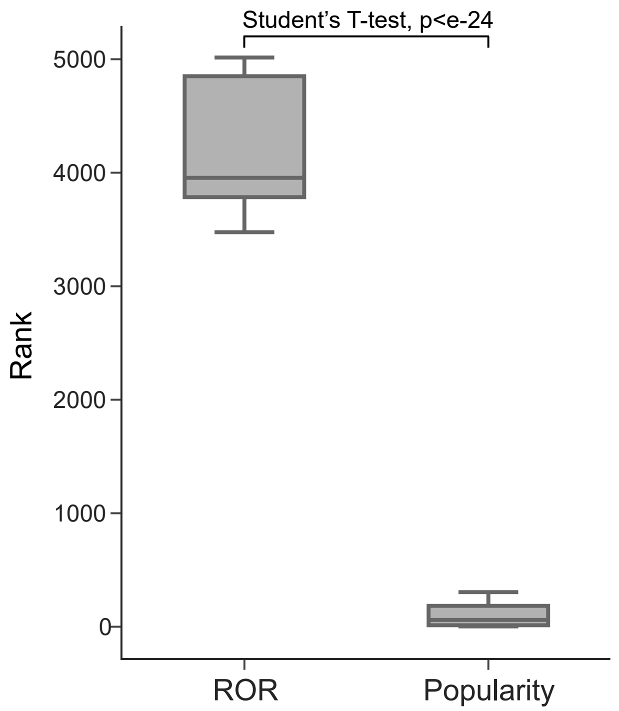


**Supplementary Figure 12: Ranks of COVID-related symptoms.** We have retrieved 17 common symptoms associated with COVID-19 from the United States CDC (https://www.cdc.gov/coronavirus/2019-ncov/symptoms-testing/symptoms.html) and WHO (https://www.who.int/emergencies/diseases/novel-coronavirus-2019/advice-for-public): dyspnoea (i.e., difficulty breathing), cough, pyrexia (i.e., fever), pneumonia, nausea, chest pain, rash, vomiting, headache, diarrhoea, respiratory symptom, respiratory tract infection, cardiovascular disorder, skin disorder, respiratory disorder, lung disorder, and dermatosis. We compare the ranks of these coronavirus-related symptoms based on their ROR (calculated in the first step of our analysis; Methods; the smaller the rank, greater the impact of the pandemic on its incidence) and popularity (*i.e.*, the number of publications; the smaller the rank, the more popular it is). We find that the ROR-based ranks significantly differ from popularity-based ranks (p-value $< 10^{-24}$; Student's T-test), indicating that our model's findings are not confounded by the popular symptoms.

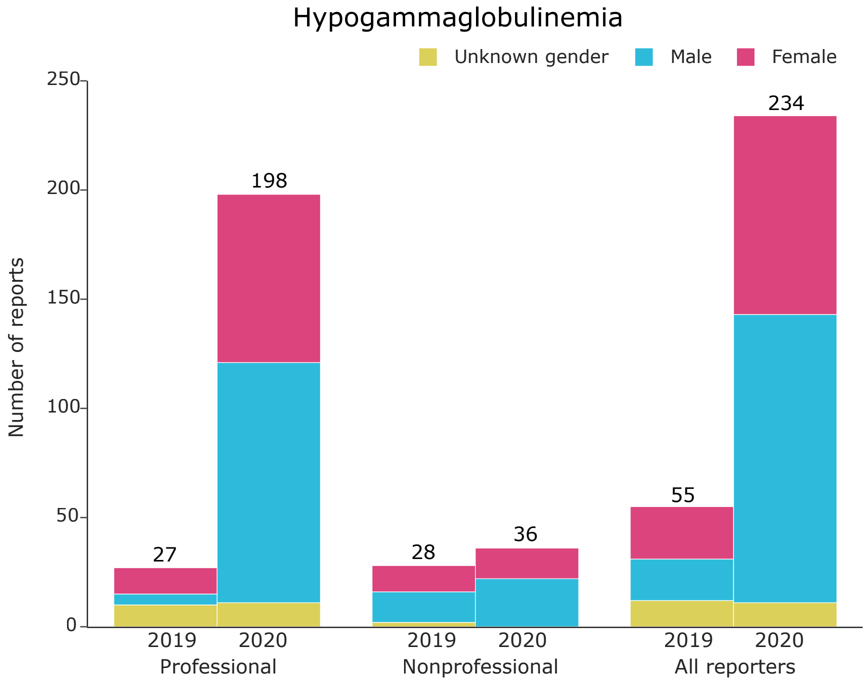


**Supplementary Figure 13: Comparison of reporter distribution of hypogammaglobulinemia before and during the pandemic.** The number of patients who have hypogammaglobulinemia as drug side effect and report to healthcare professionals has increased dramatically from 27 (physician:9, pharmacist:0, other professionals: 18) in 2019 (March 11-September 30) to 198 (physician:161, pharmacist:1, other professionals: 36) in 2020 (March 11-September 30). In contrast, the number of self-reported cases only grew slightly from 28 to 36. After omitting reports with unknown sex, the number of women who suffer from hypogammaglobulinemia as a side effect has increased from 12 to 77, despite the proportion of women dropping from 70.6% (= 12/(12+5)) to 41.2% (= 77/(77+110)). The observation indicates some underlying prevalence differences among men and women during the pandemic for hypogammaglobulinemia.

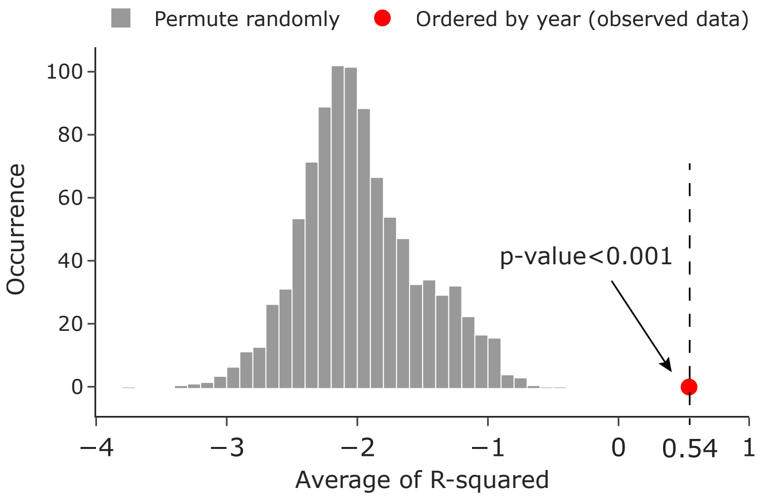


**Supplementary Figure 14: Model fitting comparison between autoregressive models trained on observed data and randomly permuted data.** The red point denotes our second-order autoregressive model whose training data are ordered by year. We fit a model for each of 105 adverse events that passed disproportionality estimation (step 1, Methods). As baselines, we train regression models based on randomly permuted training data of each adverse event for 1,000 times, and report the distribution of model fitting measurements in the grey histogram. We evaluate the model fitting by the average of R-squared scores across 105 adverse events. We found that our model (trained on observed data) is significantly different from the models trained on random permutation ($p$-value $< 0.001$; permutation test). In addition, the R-squared scores of models with random permutation are negative, implying that these fitted models are arbitrarily worse that have errors larger than the ones produced by a horizontal line. Overall, the observations show that our model can capture the historical pattern of observations even with only seven training points.

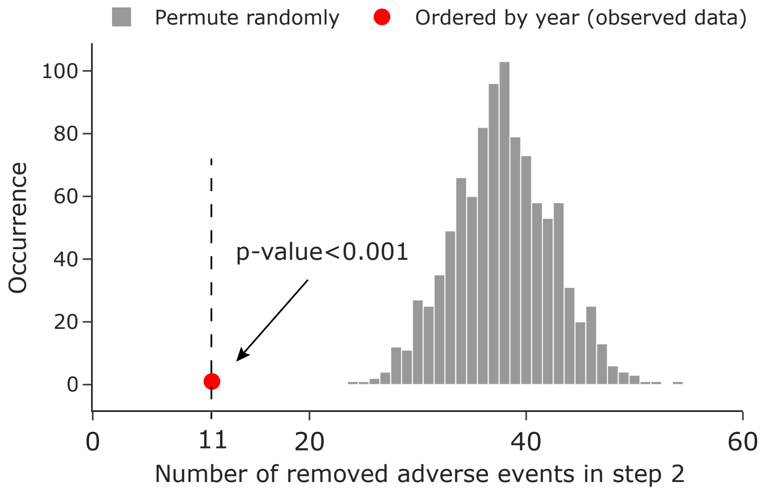


**Supplementary Figure 15: Prediction results comparison between autoregressive models trained on observed data and randomly permuted data.** The red point denotes our second-order autoregressive model whose training data are permuted by time. We fit a model for each of 105 adverse events that passed disproportionality estimation (step 1, Methods). As baselines, we train regression models based on randomly permuted training data of each adverse event for 1,000 times. Based on these fitted models, we measure the PAEAI of each adverse event and remove the ones with negative PAEAI as the abnormal reporting of them during the pandemic may be confounded by temporal trend (step 2, Methods). We present the distribution of the number of removed adverse events. In terms of prediction results, our model is significantly different from the models based on random permutation ($p$-value $< 0.001$; permutation test). In summary of Supplementary Figure 14 and Supplementary Figure 15, our AR(2) model (trained on observed data) is valid and robust by comparing model fits and prediction results with the models trained on randomly permuted reporting frequencies: indicating our model is able to discover the trajectories of adverse events' reporting frequencies and further remove the confounding of historical trends.

## Supplementary Tables

**Supplementary Table 1: Summary of adverse events identified by our approach in male patients.** Shown are 19 adverse events including 16 enriched and 3 purified during the pandemic, which are identified in the male subpopulation. The ROR represents the association between an adverse event and the pandemic (Fisher's exact test; p-value is adjusted by Bonferroni correction; Methods). The numbers in brackets show the range of lower to upper 95% confidence interval. Higher ROR indicates the adverse event has a stronger association with the pandemic. The defined PAEAI describes the extent to which an adverse event deviates from its historical trend (higher PAEAI suggests larger deviation; Methods). All identified adverse events have positive PAEAI which indicates the reporting pattern during the pandemic doesn't follow its trajectory. The adverse events are sorted in descending order of PAEAI. The 'E' and 'P' in the 'E\P' column denotes this adverse event is enriched or purified, respectively. PEx: pulmonary exacerbations.

| Adverse event | MedDRA ID | ROR (95% CI) | PAEAI (↓) | E \P |
|---|---|---|---|---|
| Hypogammaglobulinaemia | 10020983 | 22.32 [9.11,54.69] | 1.91 | E |
| Alcoholism | 10001639 | 1.90 [1.45,2.49] | 1.79 | E |
| Delusion | 10012239 | 2.96 [2.28,3.85] | 1.59 | E |
| Neutrophilia | 10029379 | 4.20 [2.92,6.04] | 1.48 | E |
| Dementia | 10012267 | 1.83 [1.41,2.38] | 1.24 | E |
| Hallucination | 10019063 | 2.79 [2.46,3.18] | 1.23 | E |
| Infective PEx of cystic fibrosis | 10070608 | 0.54 [0.44,0.67] | 1.23 | P |
| Cardiac arrest | 10007515 | 1.89 [1.66,2.16] | 1.12 | E |
| Respiratory failure | 10038695 | 1.40 [1.23,1.60] | 0.96 | E |
| Neuropathy peripheral | 10029331 | 2.32 [2.05,2.63] | 0.95 | E |
| Internal haemorrhage | 10075192 | 2.41 [1.70,3.42] | 0.90 | E |
| Respiratory arrest | 10038669 | 8.19 [5.64,11.90] | 0.80 | E |
| Headache | 10019211 | 0.83 [0.78,0.89] | 0.76 | P |
| Bradycardia | 10006093 | 1.59 [1.32,1.93] | 0.65 | E |
| Rectal haemorrhage | 10038063 | 1.86 [1.44,2.40] | 0.61 | E |
| Gastrointestinal haemorrhage | 10017955 | 1.78 [1.58,2.02] | 0.59 | E |
| Confusional state | 10010305 | 1.54 [1.38,1.73] | 0.26 | E |
| Gynaecomastia | 10018800 | 0.55 [0.43,0.70] | 0.21 | P |
| Cytokine release syndrome | 10052015 | 1.81 [1.45,2.24] | 0.08 | E |

**Supplementary Table 2: Summary of adverse events identified by our approach in young patients.** Our framework detects two adverse events (one enriched and one purified), with a significantly different reporting frequency during the pandemic in young patients. The ROR represents the association between an adverse event and the pandemic (Fisher's exact test; p-value is adjusted by Bonferroni correction; Methods). The adverse events are sorted in descending order of PAEAI. The 'E' and 'P' in the 'E\P' column denotes this adverse event is enriched or purified, respectively. More notations are the same as in Supplementary Table 1. PEx: pulmonary exacerbations.

| Adverse event | MedDRA ID | ROR (95% CI) | PAEAI (↓) | E \P |
|---|---|---|---|---|
| Infective PEx of cystic fibrosis | 10070608 | 0.48 [0.38,0.62] | 1.33 | P |
| Pyrexia | 10037660 | 1.50 [1.26,1.78] | 1.08 | E |

**Supplementary Table 3: Summary of adverse events identified by our approach in elderly patients.** Shown are 19 adverse events (18 enriched and 1 purified), identified in aged individuals. The ROR represents the association between an adverse event and the pandemic (Fisher's exact test; p-value is adjusted by Bonferroni correction; Methods). The adverse events are sorted in descending order of PAEAI. The 'E' and 'P' in the 'E\P' column denotes this adverse event is enriched or purified, respectively. Notations are the same as in Supplementary Table 1.

| Adverse event | MedDRA ID | ROR (95% CI) | PAEAI (↓) | E \P |
|---|---|---|---|---|
| Aggression | 10001488 | 2.42 [1.70,3.45] | 1.46 | E |
| Vitreous floaters | 10047654 | 8.11 [4.97,13.23] | 1.40 | E |
| Abnormal behaviour | 10061422 | 2.85 [2.03,3.98] | 1.16 | E |
| Delusion | 10012239 | 2.79 [2.09,3.72] | 1.07 | E |
| Haematuria | 10018867 | 1.98 [1.55,2.54] | 1.04 | E |
| Hallucination | 10019063 | 2.58 [2.24,2.97] | 1.01 | E |
| Cardiac arrest | 10007515 | 1.59 [1.34,1.89] | 0.99 | E |
| Internal haemorrhage | 10075192 | 2.92 [2.01,4.24] | 0.97 | E |
| Dementia | 10012267 | 1.97 [1.51,2.58] | 0.94 | E |
| General physical health deterioration | 10049438 | 1.97 [1.50,2.59] | 0.91 | E |
| Rectal haemorrhage | 10038063 | 1.88 [1.47,2.39] | 0.90 | E |
| Urinary tract infection | 10046571 | 1.29 [1.16,1.43] | 0.89 | E |
| Neuropathy peripheral | 10029331 | 1.58 [1.36,1.84] | 0.83 | E |
| Acute kidney injury | 10069339 | 1.35 [1.21,1.50] | 0.83 | E |
| Gastrointestinal haemorrhage | 10017955 | 2.00 [1.78,2.26] | 0.75 | E |
| Hypoacusis | 10048865 | 2.18 [1.78,2.66] | 0.73 | E |
| Confusional state | 10010305 | 1.51 [1.34,1.70] | 0.73 | E |
| Nausea | 10028813 | 0.85 [0.80,0.91] | 0.63 | P |
| Neoplasm progression | 10061309 | 1.92 [1.61,2.30] | 0.47 | E |

**Supplementary Table 4: Summary of COVID-related symptoms.** Shown are 17 symptoms that are strongly associated with COVID-19, retrieved from CDC of United States (https://www.cdc.gov/coronavirus/2019-ncov/symptoms-testing/symptoms.html) and WHO (https://www.who.int/emergencies/diseases/novel-coronavirus-2019/advice-for-public). The 'RF-2019' and 'RF-2020' denote the reporting frequency (per 1,000 patients) during March 11 to September 30 in 2019 and 2020, respectively. For example, the reporting frequency of dermatosis has slightly increased from 0.018 to 0.023 (which are both rounded to 0.02 in displaying only 2 significant figures). The 'Pop-rank' denotes popularity-based rank where the adverse events mentioned in more publications are assigned a smaller rank number (higher rank), while 'ROR-rank' denotes ROR-based rank where the ones with higher reporting odds ratio have smaller rank number (higher rank). The ROR is calculated in the first step of our model (Methods). All the ranks range from 1 to 7,761 as we analyze 7,761 adverse events in the first step of our model. The adverse events are sorted in ascending order of ROR-based rank. In the 'E \P' column, 'E' and 'P' denotes whether the adverse event is enriched or purified by our model, respectively; 'NS' means the adverse event is not significantly associated with the pandemic, as identified by our model. The difference of two ranks are analyzed in Supplementary Figure 12.

| Adverse event | MedDRA ID | RF-2019 | RF-2020 | Pop-rank | ROR-rank (↑) | E \P |
|---|---|---|---|---|---|---|
| Dermatosis | 10048768 | 0.02 | 0.02 | 61 | 3,476 | NS |
| Cough | 10011224 | 11.09 | 13.61 | 50 | 3,687 | E |
| Lung disorder | 10025082 | 1.40 | 1.72 | 5 | 3,687 | NS |
| Pyrexia | 10037660 | 12.50 | 14.97 | 25 | 3,757 | E |
| Dyspnoea | 10013968 | 24.51 | 29.03 | 103 | 3,794 | E |
| Chest pain | 10008479 | 7.22 | 8.48 | 291 | 3,810 | E |
| Pneumonia | 10035664 | 16.78 | 19.04 | 3 | 3,925 | E |
| Respiratory disorder | 10038683 | 1.08 | 1.23 | 8 | 3,925 | NS |
| Cardiovascular disorder | 10007649 | 0.29 | 0.33 | 15 | 3,955 | NS |
| Skin disorder | 10040831 | 1.20 | 1.36 | 60 | 3,955 | NS |
| Respiratory tract infection | 10062352 | 0.87 | 0.97 | 7 | 4,002 | NS |
| Respiratory symptom | 10075535 | 0.13 | 0.14 | 51 | 4,069 | NS |
| Diarrhoea | 10012735 | 38.26 | 39.18 | 131 | 4,843 | NS |
| Headache | 10019211 | 33.31 | 33.31 | 171 | 4,873 | NS |
| Vomiting | 10047700 | 20.42 | 19.46 | 223 | 4,970 | NS |
| Rash | 10037844 | 23.16 | 21.84 | 305 | 4,993 | NS |
| Nausea | 10028813 | 42.43 | 39.54 | 247 | 5,015 | P |

**Supplementary Table 5: Summary statistics of dataset.** Shown are number of reports by year from 2013 to 2020 (2020 only has the first three quarters). The columns contain the number of reports in various situations. '#-original': all reports included in raw FAERS dataset; '#-unique': unique reports after removing duplication; '#-US': reports occurred in the USA; '#-period': reports submitted from March 11 to September 30; '#-professional': reports submitted by healthcare professionals.

| Year | #-original | #-unique | #-US | #-period | #-professionals |
|---|---|---|---|---|---|
| 2013 | 812,596 | 767,864 | 509,243 | 249,593 | 89,334 |
| 2014 | 903,174 | 810,139 | 527,902 | 286,491 | 97,813 |
| 2015 | 1,319,905 | 1,222,971 | 875,144 | 530,604 | 179,400 |
| 2016 | 1,004,885 | 1,148,123 | 768,166 | 418,839 | 169,279 |
| 2017 | 1,369,547 | 1,211,753 | 869,603 | 488,700 | 210,826 |
| 2018 | 1,684,852 | 1,429,021 | 985,988 | 561,913 | 244,155 |
| 2019 | 1,727,296 | 1,472,966 | 988,027 | 570,532 | 221,019 |
| 2020 | 1,321,221 | 1,227,899 | 814,785 | 596,508 | 211,219 |

## Supplementary references


1. Czeisler, M. É. *et al.* Delay or avoidance of medical care because of covid-19–related concerns—united states, june 2020. *Morbidity and Mortality Weekly Report* **69**, 1250 (2020).

2. Goldman, J. D. *et al.* Remdesivir for 5 or 10 days in patients with severe covid-19. *New England Journal of Medicine* **383**, 1827–1837 (2020).

3. Brown, E. G., Wood, L. & Wood, S. The medical dictionary for regulatory activities (MedDRA). *Drug Safety* **20**, 109–117 (1999).

4. Tatonetti, N. P., Patrick, P. Y., Daneshjou, R. & Altman, R. B. Data-driven prediction of drug effects and interactions. *Science Translational Medicine* **4**, 125ra31–125ra31 (2012).

5. Wishart, D. S. *et al.* Drugbank 5.0: a major update to the DrugBank database for 2018. *Nucleic Acids Research* **46**, D1074–D1082 (2018).

6. WHO. ATC/DDD classification. *WHO Drug Information* **33**, 226–232 (2019).

7. WHO. Men, ageing and health: achieving health across the life span. Tech. Rep., World Health Organization (2001).
